# Supplementary material for: Survival time and prognostic factors in dogs clinically diagnosed with haemangiosarcoma in UK first opinion practice
Source: PLoS One. 2025 Jun 6;20(6):e0316066. doi: 10.1371/journal.pone.0316066 (PMC12143555; doi:10.1371/journal.pone.0316066)
Supplement: S1 Table — ‘Variable’ and ‘information from EPRs’ columns show what information was captured from EPRs and what it was transformed or grouped into for regression analyses (‘Grouped variables’). (DOCX) [file pone.0316066.s001.docx]

# Supplementary material - Survival time and prognostic factors in dogs clinically diagnosed with haemangiosarcoma in UK first opinion practice

**Table S1-** Complete list of information coded on clinical signs, diagnostic tests, surgical and medical management options and tumour size and location from clinical records of haemangiosarcoma cases diagnosed in 2019 in VetCompass EPRs. ‘Variable’ and ‘information from EPRs’ columns show what information was captured from EPRs and what it was transformed or grouped into for regression a1lyses (‘Grouped variables’).

| **Variable** | **Information from EPRs (alphabetical order)** | **Grouped variables** |
| --- | --- | --- |
| **Clinical signs** | Record of:  Abdominal distension, Abdominal effusion (not haemabdomen or no abdominocentesis), Abdominal pain, anorexia/hyporexia, arrhythmia, bounding pulses, bruising, coat changes, collapse, coughing, diarrhoea, dyspnoea, epistaxis, exercise intolerance, exophthalmos, facial asymmetry, generalised pain, haemabdomen, haematochezia, haematuria or haemaglobinuria, haemoptysis, haemorrhagic ocular conditions/hyphaema, halitosis, heart murmur, hypersalivation, hypodipsia, hypothermia, icterus, increased respiratory effort, lameness, lethargy, localised pain to neck/mouth/thoracic, melae1, muffled heart auscultation, neurological deficits/nystagmus/seizures/ataxia/anisocoria, oedema peripheral, organomegaly hepatic or splenic, pale mucus membranes, palpable Abdominal mass, polyuria/polydipsia, pericardial effusion, peripheral lymphadenopathy, petechiae, pica, pleural effusion, polyphagia, pulmo1ry crackles, pyrexia, sneezing, soft tissue mass, stertor/stridor, stranguria, tachycardia, tachypnoea, tenesmus, ulcerated mass, Urinary incontinence, vocalisation, vomiting, weakness, weight loss, no clinical signs | - Clinical signs (CS):   - Haematological   - Cardiac   - Respiratory   - Gastrointestinal   - Urinary   - Mass specific   - Other specific   - Non specific   - No CS   - Effusion present |
| **Diagnostic tests performed** | Record of:  Abdominal radiography, Abdominal ultrasound, abdominocentesis, aspirate cytology, biopsy histopathology, blood tests (complete blood count, biochemistry or electrolyte), coagulation profile, coombs test, cerebrospi1l fluid sampling, CT/MRI, electrocardiogram, echocardiography, ocular ultrasound, pericardiocentesis, post-mortem, radiography local to mass, rhinoscopy, thoracic radiography, thoracic ultrasound, thoracocentesis, uri1lysis, ultrasound of exter1l mass | - Diagnostic test groups:   - Imaging   - Laboratory tests   - Sampling   - Cardiac diagnostics   - Abdominal diagnostics   - No diagnostics done |
| **Surgical management options** | Record of:  Exploratory laparotomy, splenectomy, liver lobectomy, mass removal, incisio1l biopsy, limb/digit amputation, enterectomy, nephrectomy, enucleation, sinusotomy, unrecorded surgical procedure | - Did they have any surgery performed? - Did they have surgery and medical management performed? |
| **Medical management options** | Record of:  Anti-seizure medications, auto-transfusion, blood product transfusion, CBD oil, chemotherapy, coriolus versicolor (turkey tail mushroom), diuretics, doxorubicin, epirubicin, ferrous sulphate, liver support supplement, omega 3 supplement, palliative a1lgesia, palliative steroids, pimobendane, propranolol, thalidomide, tranexamic acid, vincristine/cyclophosphamide, yun1n baiyao | - Did they have any medical management? - Type of medical management:   - Cardiac   - Alter1tive supplementation   - Transfusion   - Haemostatic   - Palliative |
| **Tumour specifics** | Tumour location:  Abdominal unspecified, aorta, bone, brain, cardiac, conjunctiva/third eyelid,cutaneous, frontal sinus, hepatic, intestine, intramuscular, kidney, lung, lymph node, multisite, 1sal, ocular, omentum, oral/gingival/tongue, pancreas, pleural space, prepuce, prostate, retrobulbar, retroperitoneum splenic, subcutaneous, urethral, Urinary bladder  Metastases location:  Brain, cardiac, cutaneous, intestines, kidney, liver, lungs, lymph node, 1sal, omentum pancreas, peritoneum, pleural space, spleen, subcutaneous, Urinary bladder, no location recorded  Tumour size (cm) | - Any tumour location:   - Splenic   - Hepatic   - Cardiac   - Cutaneous - Presence of metastases? - Metastatic tumour location:   - Abdominal metastases   - Thoracic metastases   - Cranial metastases   - Soft tissue metastases   - Lymphatic metastases   - No location specified - Main interest:   - Cardiac   - Splenic   - Hepatic   Tumour size  Maximum tumour size (quartiles,terciles and continuous) |
