# Supplementary material for: Survival time and prognostic factors in dogs clinically diagnosed with haemangiosarcoma in UK first opinion practice
Source: PLoS One. 2025 Jun 6;20(6):e0316066. doi: 10.1371/journal.pone.0316066 (PMC12143555; doi:10.1371/journal.pone.0316066)
Supplement: S2 Table — Percentages shown are column percentages. (DOCX) [file pone.0316066.s002.docx]

# Supplementary material - Survival time and prognostic factors in dogs clinically diagnosed with haemangiosarcoma in UK first opinion practice

**Table S2-**Descriptive statistics and univariable logistic regression results of haemangiosarcoma cases diagnosed in first-opinion practices in VetCompass in 2019 with <1 day survival (‘Day 0’, n=381) and ≥1 day of survival post presentation (‘Day 1’, n=407). Percentages shown are column percentages.

| Variable | Category | Day 1 | Day 0 | OR (95%CI) | Wald p-value | LRT p-value |
| --- | --- | --- | --- | --- | --- | --- |
| Corporate group | Group A | 84 (22.1) | 80 (19.7) | 1 (1 - 1) |  | 0.69 |
|  | Group B | 129 (33.9) | 158 (38.9) | 0.78 (0.53 - 1.14) | 0.20 |  |
|  | Group C | 8 (2.1) | 7 (1.7) | 1.09 (0.38 - 3.14) | 0.87 |  |
|  | Group D | 109 (28.7) | 111 (27.3) | 0.94 (0.62 - 1.4) | 0.74 |  |
|  | Group E | 50 (13.2) | 50 (12.3) | 0.95 (0.58 - 1.57) | 0.85 |  |
| Sex | female | 176 (46.3) | 183 (45.1) | 1 (1 - 1) |  | 0.88 |
|  | male | 202 (53.2) | 220 (54.2) | 0.95 (0.72 - 1.27) | 0.75 |  |
|  | unrecorded | 2 (0.5) | 3 (0.7) | 0.69 (0.11 - 4.2) | 0.69 |  |
| Sex-neuter status | female entire | 42 (11.1) | 33 (8.1) | 1 (1 - 1) |  | 0.54 |
|  | female neutered | 134 (35.3) | 150 (36.9) | 0.7 (0.42 - 1.17) | 0.17 |  |
|  | male entire | 58 (15.3) | 72 (17.7) | 0.63 (0.36 - 1.12) | 0.12 |  |
|  | male neutered | 144 (37.9) | 148 (36.5) | 0.76 (0.46 - 1.27) | 0.30 |  |
|  | unrecorded entire | 2 (0.5) | 2 (0.5) | 0.79 (0.1 - 5.88) | 0.81 |  |
|  | unrecorded neutered |  | 1 (0.2) | 0 (0 - Inf) | 0.98 |  |
| Neuter | Entire | 102 (26.8) | 107 (26.4) | 1 (1 - 1) |  | 0.88 |
|  | Neutered | 278 (73.2) | 299 (73.6) | 0.98 (0.71 - 1.34) | 0.88 |  |
| Age at diagnosis (quartiles, years) | 11-13 | 131 (34.5) | 106 (26.1) | 1 (1 - 1) |  | 0.07 |
|  | 13-15 | 48 (12.6) | 46 (11.3) | 0.84 (0.52 - 1.36) | 0.49 |  |
|  | 5-7 | 17 (4.5) | 19 (4.7) | 0.72 (0.36 - 1.46) | 0.37 |  |
|  | 7-9 | 64 (16.8) | 87 (21.4) | 0.6 (0.39 - 0.9) | 0.01 |  |
|  | 9-11 | 113 (29.7) | 144 (35.5) | 0.64 (0.45 - 0.91) | 0.01 |  |
|  | Above 15 | 7 (1.8) | 4 (1.0) | 1.42 (0.4 - 4.97) | 0.59 |  |
| Patient neutering status prior to presentation | Neutered prior to diagnosis | 276 (72.6) | 301 (74.1) | 1 (1 - 1) |  | 0.53 |
|  | Entire | 103 (27.1) | 102 (25.1) | 1.1 (0.8 - 1.51) | 0.55 |  |
|  | Neutered after diagnosis | 1 (0.3) | 3 (0.7) | 0.36 (0.04 - 3.51) | 0.38 |  |
| Presumptive diagnosis | Definitive | 15 (3.9) | 297 (73.2) | 1 (1 - 1) |  | <0.001 |
|  | Presumptive only | 365 (96.1) | 109 (26.8) | 66.3 (37.83 - 116.2) | <0.001 |  |
| Haematological clinical signs present | No haematological signs | 197 (51.8) | 291 (71.7) | 1 (1 - 1) |  | <0.001 |
|  | Haematological signs present | 183 (48.2) | 115 (28.3) | 2.35 (1.75 - 3.16) | <0.001 |  |
| Cardiac clinical signs present | No cardiac signs | 238 (62.6) | 345 (85.0) | 1 (1 - 1) |  | <0.001 |
|  | Cardiac signs present | 142 (37.4) | 61 (15.0) | 3.37 (2.4 - 4.75) | <0.001 |  |
| Respiratory clinical signs present | No respiratory signs | 307 (80.8) | 374 (92.1) | 1 (1 - 1) |  | <0.001 |
|  | Respiratory signs present | 73 (19.2) | 32 (7.9) | 2.78 (1.79 - 4.32) | <0.001 |  |
| Gastrointestinal clinical signs present | No gastrointestinal signs | 275 (72.4) | 347 (85.5) | 1 (1 - 1) |  | <0.001 |
|  | Gastrointestinal signs present | 105 (27.6) | 59 (14.5) | 2.25 (1.57 - 3.21) | <0.001 |  |
| Urinary clinical signs present | No Urinary signs | 325 (85.5) | 373 (91.9) | 1 (1 - 1) |  | <0.01 |
|  | Urinary signs present | 55 (14.5) | 33 (8.1) | 1.91 (1.21 - 3.02) | <0.01 |  |
| Non-specific clinical signs present | No non-specific signs | 31 (8.2) | 147 (36.2) | 1 (1 - 1) |  | <0.001 |
|  | Non-specific signs present | 349 (91.8) | 259 (63.8) | 6.39 (4.2 - 9.72) | <0.001 |  |
| Other clinical signs present | No other signs | 31 (8.2) | 147 (36.2) | 1 (1 - 1) |  | <0.001 |
|  | Other signs present | 349 (91.8) | 259 (63.8) | 6.39 (4.2 - 9.72) | <0.001 |  |
| Mass associated clinical signs present | No mass associated signs | 296 (77.9) | 220 (54.2) | 1 (1 - 1) |  | <0.001 |
|  | Mass associated signs present | 84 (22.1) | 186 (45.8) | 0.34 (0.25 - 0.46) | <0.001 |  |
| No clinical signs present | Clinical signs present | 378 (99.5) | 380 (93.6) | 1 (1 - 1) |  | <0.001 |
|  | No clinical signs present | 2 (0.5) | 26 (6.4) | 0.08 (0.02 - 0.33) | <0.001 |  |
| Imaging performed | No imaging performed | 70 (18.4) | 95 (23.4) | 1 (1 - 1) |  | 0.09 |
|  | Imaging performed | 310 (81.6) | 311 (76.6) | 1.35 (0.96 - 1.91) | 0.09 |  |
| Samples taken | No samples taken | 264 (69.5) | 102 (25.1) | 1 (1 - 1) |  | <0.001 |
|  | Samples taken | 116 (30.5) | 304 (74.9) | 0.15 (0.11 - 0.2) | <0.001 |  |
| Laboratory tests performed | No lab tests performed | 173 (45.5) | 131 (32.3) | 1 (1 - 1) |  | <0.001 |
|  | Lab tests performed | 207 (54.5) | 275 (67.7) | 0.57 (0.43 - 0.76) | <0.001 |  |
| Cardiac diagnostics performed | No cardiac diagnostics | 343 (90.3) | 367 (90.4) | 1 (1 - 1) |  | 0.95 |
|  | Cardiac diagnostics performed | 37 (9.7) | 39 (9.6) | 1.02 (0.63 - 1.63) | 0.95 |  |
| Abdominal diagnostics performed | No Abdominal diagnostics | 73 (19.2) | 143 (35.2) | 1 (1 - 1) |  | <0.001 |
|  | Abdominal diagnostics performed | 307 (80.8) | 263 (64.8) | 2.29 (1.65 - 3.17) | <0.001 |  |
| No diagnostics performed | Diagnostics performed | 347 (91.3) | 398 (98.0) | 1 (1 - 1) |  | <0.001 |
|  | No diagnostics performed | 33 (8.7) | 8 (2.0) | 4.73 (2.16 - 10.38) | <0.001 |  |
| Any surgical management performed | No surgery | 336 (88.4) | 126 (31.0) | 1 (1 - 1) |  | <0.001 |
|  | Surgery | 44 (11.6) | 280 (69.0) | 0.06 (0.04 - 0.09) | <0.001 |  |
| Any medical management performed | No medicine | 361 (95.0) | 268 (66.0) | 1 (1 - 1) |  | <0.001 |
|  | Medicine | 19 (5.0) | 138 (34.0) | 0.1 (0.06 - 0.17) | <0.001 |  |
| Any cardiac medical management performed | No cardiac medicine | 379 (99.7) | 403 (99.3) | 1 (1 - 1) |  | 0.37 |
|  | Cardiac medicine | 1 (0.3) | 3 (0.7) | 0.35 (0.04 - 3.42) | 0.37 |  |
| Any alter1tive medical management performed | No alter1tive medicine | 379 (99.7) | 397 (97.8) | 1 (1 - 1) |  | 0.04 |
|  | Alter1tive medicine | 1 (0.3) | 9 (2.2) | 0.12 (0.01 - 0.92) | 0.04 |  |
| Any transfusion medical management performed | No transfusion | 375 (98.7) | 380 (93.6) | 1 (1 - 1) |  | <0.001 |
|  | Transfusion | 5 (1.3) | 26 (6.4) | 0.19 (0.07 - 0.51) | <0.001 |  |
| Any haemostatic medical management performed | No haemostatic medicine | 370 (97.4) | 372 (91.6) | 1 (1 - 1) |  | <0.001 |
|  | Haemostatic medicine | 10 (2.6) | 34 (8.4) | 0.3 (0.14 - 0.61) | <0.001 |  |
| Any palliative medical management performed | No palliative medicine | 370 (97.4) | 327 (80.5) | 1 (1 - 1) |  | <0.001 |
|  | Palliative medicine | 10 (2.6) | 79 (19.5) | 0.11 (0.06 - 0.22) | <0.001 |  |
| No medical management performed | Medicine | 19 (5.0) | 138 (34.0) | 1 (1 - 1) |  | <0.001 |
|  | No medicine | 361 (95.0) | 268 (66.0) | 9.78 (5.9 - 16.21) | <0.001 |  |
| No surgical management performed | Surgery | 44 (11.6) | 280 (69.0) | 1 (1 - 1) |  | <0.001 |
|  | No surgery | 336 (88.4) | 126 (31.0) | 16.97 (11.63 - 24.77) | <0.001 |  |
| Medical and surgical management performed | No medicine and surgery | 375 (98.7) | 317 (78.1) | 1 (1 - 1) |  | <0.001 |
|  | Medicine and surgery | 5 (1.3) | 89 (21.9) | 0.05 (0.02 - 0.12) | <0.001 |  |
| No medical or surgical management performed | Medicine or surgery | 58 (15.3) | 329 (81.0) | 1 (1 - 1) |  | <0.001 |
|  | No medicine or surgery | 322 (84.7) | 77 (19.0) | 23.72 (16.32 - 34.47) | <0.001 |  |
| Chemotherapy dose | High dose chemotherapy | 0 (0.0) | 27 (6.7) | 1 (1 - 1) |  | 0.97 |
|  | Metronomic chemotherapy | 1 (0.3) | 10 (2.5) | 1565136.04 (0 - Inf) | 0.97 |  |
|  | No chemotherapy | 379 (99.7) | 369 (90.9) | 16075516.53 (0 - Inf) | 0.97 |  |
| Visited a referral centre | No referral centre | 366 (96.3) | 328 (80.8) | 1 (1 - 1) |  | <0.001 |
|  | Visited referral centre | 14 (3.7) | 78 (19.2) | 0.16 (0.09 - 0.29) | <0.001 |  |
| Abdominal metastases present | No Abdominal metastases | 321 (84.5) | 349 (86.0) | 1 (1 - 1) |  | 0.56 |
|  | Abdominal metastases | 59 (15.5) | 57 (14.0) | 1.13 (0.76 - 1.67) | 0.56 |  |
| Thoracic metastases present | No thoracic metastases | 359 (94.5) | 384 (94.6) | 1 (1 - 1) |  | 0.94 |
|  | Thoracic metastases | 21 (5.5) | 22 (5.4) | 1.02 (0.55 - 1.89) | 0.94 |  |
| Cranial metastases present | No cranial metastases | 379 (99.7) | 404 (99.5) | 1 (1 - 1) |  | 0.60 |
|  | Cranial metastases | 1 (0.3) | 2 (0.5) | 0.53 (0.05 - 5.9) | 0.60 |  |
| Soft tissue metastases present | No soft tissue metastases | 376 (98.9) | 390 (96.1) | 1 (1 - 1) |  | 0.02 |
|  | Soft tissue metastases | 4 (1.1) | 16 (3.9) | 0.26 (0.09 - 0.78) | 0.02 |  |
| Lymph node metastases present | No lymph node metastases | 377 (99.2) | 402 (99.0) | 1 (1 - 1) |  | 0.77 |
|  | Lymph node metastases | 3 (0.8) | 4 (1.0) | 0.8 (0.18 - 3.6) | 0.77 |  |
| Unspecified metastases present | No unspecified metastases | 380 (100.0) | 401 (98.8) | 1 (1 - 1) |  | 0.97 |
|  | Unspecified metastases | 0 (0.0) | 5 (1.2) | 0 (0 - Inf) | 0.97 |  |
| Any metastases present | No metastases present | 301 (79.2) | 316 (77.8) | 1 (1 - 1) |  | 0.64 |
|  | Metastases present | 79 (20.8) | 90 (22.2) | 0.92 (0.66 - 1.3) | 0.64 |  |
| Cardiac interest location | Cardiac | 57 (15.0) | 17 (4.2) | 1 (1 - 1) |  | <0.001 |
|  | No location specified | 41 (10.8) | 16 (3.9) | 0.76 (0.35 - 1.69) | 0.51 |  |
|  | Non cardiac cutaneous | 12 (3.2) | 124 (30.5) | 0.03 (0.01 - 0.06) | <0.001 |  |
|  | Non cardiac visceral | 270 (71.1) | 249 (61.3) | 0.32 (0.18 - 0.57) | <0.001 |  |
| Splenic interest location | No location specified | 41 (10.8) | 16 (3.9) | 1 (1 - 1) |  | <0.001 |
|  | Non splenic cutaneous | 12 (3.2) | 124 (30.5) | 0.04 (0.02 - 0.09) | <0.001 |  |
|  | Non splenic visceral | 68 (17.9) | 45 (11.1) | 0.59 (0.3 - 1.18) | 0.13 |  |
|  | Splenic | 259 (68.2) | 221 (54.4) | 0.46 (0.25 - 0.84) | 0.01 |  |
| Hepatic interest location | Hepatic | 96 (25.3) | 43 (10.6) | 1 (1 - 1) |  | <0.001 |
|  | No location specified | 41 (10.8) | 16 (3.9) | 1.15 (0.58 - 2.27) | 0.64 |  |
|  | Non hepatic cutaneous | 12 (3.2) | 124 (30.5) | 0.04 (0.02 - 0.09) | <0.001 |  |
|  | Non hepatic visceral | 231 (60.8) | 223 (54.9) | 0.46 (0.31 - 0.7) | <0.001 |  |
| Any haemangiosarcoma cutaneous location | No cutaneous location | 357 (93.9) | 268 (66.0) | 1 (1 - 1) |  | <0.001 |
|  | Cutaneous location | 23 (6.1) | 138 (34.0) | 0.13 (0.08 - 0.2) | <0.001 |  |
| Any haemangiosarcoma hepatic location | No hepatic location | 257 (67.6) | 331 (81.5) | 1 (1 - 1) |  | <0.001 |
|  | Hepatic location | 123 (32.4) | 75 (18.5) | 2.11 (1.52 - 2.94) | <0.001 |  |
| Any haemangiosarcoma splenic location | No splenic location | 120 (31.6) | 177 (43.6) | 1 (1 - 1) |  | <0.001 |
|  | Splenic location | 260 (68.4) | 229 (56.4) | 1.67 (1.25 - 2.24) | <0.001 |  |
| Any haemangiosarcoma cardiac location | No cardiac location | 323 (85.0) | 389 (95.8) | 1 (1 - 1) |  | <0.001 |
|  | Cardiac location | 57 (15.0) | 17 (4.2) | 4.04 (2.3 - 7.08) | <0.001 |  |
| Clinic postcode urban-rural status | Missing |  |  | 1 (1 - 1) |  | 0.71 |
|  | Mixed urban/rural | 180 (50.6) | 193 (51.2) | 1.13 (0.63 - 2.01) | 0.68 |  |
|  | Rural | 51 (14.3) | 63 (16.7) | 0.98 (0.51 - 1.88) | 0.95 |  |
|  | Urban | 125 (35.1) | 121 (32.1) | 1.25 (0.69 - 2.27) | 0.46 |  |
| Clinic postcode IMD quintile | 1 (most deprived) | 36 (10.1) | 48 (12.7) | 1 (1 - 1) |  | 0.68 |
|  | 2 | 73 (20.5) | 82 (21.8) | 1.19 (0.7 - 2.03) | 0.53 |  |
|  | 3 | 74 (20.8) | 78 (20.7) | 1.26 (0.74 - 2.16) | 0.39 |  |
|  | 4 | 77 (21.6) | 85 (22.5) | 1.21 (0.71 - 2.05) | 0.48 |  |
|  | 5 (least deprived) | 96 (27.0) | 84 (22.3) | 1.52 (0.9 - 2.57) | 0.11 |  |
|  | missing |  |  | 1.1 (0.55 - 2.2) | 0.78 |  |
| Top 20 VetCompass breeds | Border Collie | 13 (3.5) | 13 (3.2) | 1 (1 - 1) |  | 0.73 |
|  | Border Terrier | 2 (0.5) | 1 (0.2) | 2 (0.16 - 24.87) | 0.59 |  |
|  | Cavalier King Charles Spaniel | 1 (0.3) | 4 (1.0) | 0.25 (0.02 - 2.55) | 0.24 |  |
|  | Cockapoo | 2 (0.5) | 1 (0.2) | 2 (0.16 - 24.87) | 0.59 |  |
|  | Crossbreed | 82 (22.2) | 95 (23.7) | 0.86 (0.38 - 1.97) | 0.72 |  |
|  | English Cocker Spaniel | 16 (4.3) | 17 (4.2) | 0.94 (0.34 - 2.63) | 0.91 |  |
|  | English Springer Spaniel | 12 (3.3) | 9 (2.2) | 1.33 (0.42 - 4.24) | 0.63 |  |
|  | French Bulldog | 1 (0.3) | 1 (0.2) | 1 (0.06 - 17.75) | 1 |  |
|  | German Shepherd Dog | 43 (11.7) | 41 (10.2) | 1.05 (0.44 - 2.53) | 0.91 |  |
|  | Golden Retriever | 7 (1.9) | 16 (4.0) | 0.44 (0.14 - 1.42) | 0.17 |  |
|  | Jack Russell Terrier | 6 (1.6) | 12 (3.0) | 0.5 (0.14 - 1.74) | 0.27 |  |
|  | Labrador Retriever | 48 (13.0) | 48 (12.0) | 1 (0.42 - 2.38) | 1 |  |
|  | Miniature Dachshund | 2 (0.5) | 2 (0.5) | 1 (0.12 - 8.21) | 1 |  |
|  | missing |  |  | 2.2 (0.6 - 8.13) | 0.24 |  |
|  | Other breed | 104 (28.2) | 112 (27.9) | 0.93 (0.41 - 2.1) | 0.86 |  |
|  | Staffordshire Bull Terrier | 24 (6.5) | 19 (4.7) | 1.26 (0.48 - 3.35) | 0.63 |  |
|  | West Highland White Terrier | 5 (1.4) | 8 (2.0) | 0.62 (0.16 - 2.43) | 0.4 |  |
|  | Yorkshire Terrier | 1 (0.3) | 2 (0.5) | 0.5 (0.04 - 6.22) | 0.59 |  |
| Breeds with >=5 cases | Beagle | 2 (0.5) | 12 (3.0) | 1 (1 - 1) |  | 0.03 |
|  | Bichon Frise | 8 (2.1) | 5 (1.2) | 9.6 (1.48 - 62.16) | 0.02 |  |
|  | Border Collie | 13 (3.4) | 13 (3.2) | 6 (1.12 - 32.28) | 0.04 |  |
|  | Boxer | 9 (2.4) | 15 (3.7) | 3.6 (0.65 - 19.9) | 0.14 |  |
|  | Cavalier King Charles Spaniel | 1 (0.3) | 4 (1.0) | 1.5 (0.11 - 21.31) | 0.76 |  |
|  | Crossbreed | 82 (21.6) | 95 (23.4) | 5.18 (1.13 - 23.82) | 0.03 |  |
|  | Dogue de Bordeaux | 5 (1.3) | 1 (0.2) | 30 (2.19 - 411.01) | 0.01 |  |
|  | English Cocker Spaniel | 16 (4.2) | 17 (4.2) | 5.65 (1.09 - 29.27) | 0.04 |  |
|  | English Springer Spaniel | 12 (3.2) | 9 (2.2) | 8 (1.42 - 45.06) | 0.02 |  |
|  | Flat Coated Retriever | 2 (0.5) | 6 (1.5) | 2 (0.22 - 17.89) | 0.53 |  |
|  | German Shepherd Dog | 43 (11.3) | 41 (10.1) | 6.29 (1.33 - 29.85) | 0.02 |  |
|  | Golden Retriever | 7 (1.8) | 16 (3.9) | 2.62 (0.46 - 14.97) | 0.28 |  |
|  | Hungarian Vizsla | 3 (0.8) | 8 (2.0) | 2.25 (0.3 - 16.63) | 0.43 |  |
|  | Jack Russell Terrier | 6 (1.6) | 12 (3.0) | 3 (0.5 - 17.95) | 0.23 |  |
|  | Labradoodle | 5 (1.3) | 5 (1.2) | 6 (0.86 - 41.9) | 0.07 |  |
|  | Labrador Retriever | 48 (12.6) | 48 (11.8) | 6 (1.27 - 28.26) | 0.02 |  |
|  | Lurcher | 9 (2.4) | 5 (1.2) | 10.8 (1.69 - 68.94) | 0.01 |  |
|  | Miniature Sch1uzer | 4 (1.1) | 8 (2.0) | 3 (0.44 - 20.44) | 0.26 |  |
|  | Other breed | 68 (17.9) | 57 (14.0) | 7.16 (1.54 - 33.32) | 0.01 |  |
|  | Rottweiler | 8 (2.1) | 2 (0.5) | 24 (2.78 - 206.97) | <0.01 |  |
|  | Staffordshire Bull Terrier | 24 (6.3) | 19 (4.7) | 7.58 (1.51 - 38.05) | 0.01 |  |
|  | West Highland White Terrier | 5 (1.3) | 8 (2.0) | 3.75 (0.58 - 24.28) | 0.17 |  |
| Breeds with >=10 cases | Beagle | 2 (0.5) | 12 (3.0) | 1 (1 - 1) |  | 0.08 |
|  | Bichon Frise | 8 (2.1) | 5 (1.2) | 9.6 (1.48 - 62.16) | 0.02 |  |
|  | Border Collie | 13 (3.4) | 13 (3.2) | 6 (1.12 - 32.28) | 0.04 |  |
|  | Boxer | 9 (2.4) | 15 (3.7) | 3.6 (0.65 - 19.9) | 0.14 |  |
|  | Crossbreed | 82 (21.6) | 95 (23.4) | 5.18 (1.13 - 23.82) | 0.03 |  |
|  | English Cocker Spaniel | 16 (4.2) | 17 (4.2) | 5.65 (1.09 - 29.27) | 0.04 |  |
|  | English Springer Spaniel | 12 (3.2) | 9 (2.2) | 8 (1.42 - 45.06) | 0.02 |  |
|  | German Shepherd Dog | 43 (11.3) | 41 (10.1) | 6.29 (1.33 - 29.85) | 0.02 |  |
|  | Golden Retriever | 7 (1.8) | 16 (3.9) | 2.62 (0.46 - 14.97) | 0.28 |  |
|  | Hungarian Vizsla | 3 (0.8) | 8 (2.0) | 2.25 (0.3 - 16.63) | 0.43 |  |
|  | Jack Russell Terrier | 6 (1.6) | 12 (3.0) | 3 (0.5 - 17.95) | 0.23 |  |
|  | Labradoodle | 5 (1.3) | 5 (1.2) | 6 (0.86 - 41.9) | 0.07 |  |
|  | Labrador Retriever | 48 (12.6) | 48 (11.8) | 6 (1.27 - 28.26) | 0.02 |  |
|  | Lurcher | 9 (2.4) | 5 (1.2) | 10.8 (1.69 - 68.94) | 0.01 |  |
|  | Miniature Sch1uzer | 4 (1.1) | 8 (2.0) | 3 (0.44 - 20.44) | 0.26 |  |
|  | Other breed | 76 (20.0) | 68 (16.7) | 6.71 (1.45 - 31.04) | 0.01 |  |
|  | Rottweiler | 8 (2.1) | 2 (0.5) | 24 (2.78 - 206.97) | <0.01 |  |
|  | Staffordshire Bull Terrier | 24 (6.3) | 19 (4.7) | 7.58 (1.51 - 38.05) | 0.01 |  |
|  | West Highland White Terrier | 5 (1.3) | 8 (2.0) | 3.75 (0.58 - 24.28) | 0.16 |  |
| Ancestral group breed | Ancient group | 4 (1.1) | 3 (0.7) | 1 (1 - 1) |  | 0.59 |
|  | Crossbreed | 82 (21.6) | 95 (23.4) | 0.65 (0.14 - 2.98) | 0.58 |  |
|  | Herding sighthound group | 20 (5.3) | 19 (4.7) | 0.79 (0.16 - 4) | 0.77 |  |
|  | Mastiff terrier group | 106 (27.9) | 129 (31.8) | 0.62 (0.13 - 2.81) | 0.53 |  |
|  | Modern group | 91 (23.9) | 92 (22.7) | 0.74 (0.16 - 3.41) | 0.70 |  |
|  | Mountain group | 9 (2.4) | 4 (1.0) | 1.69 (0.25 - 11.34) | 0.59 |  |
|  | No ancestral group | 68 (17.9) | 64 (15.8) | 0.8 (0.17 - 3.7) | 0.77 |  |
| Genotype group breed | Category 1 | 46 (12.1) | 41 (10.1) | 1 (1 - 1) |  | 0.6781 |
|  | Category 2 | 37 (9.7) | 49 (12.1) | 0.67 (0.37 - 1.23) | 0.19 |  |
|  | Category 3 | 49 (12.9) | 59 (14.5) | 0.74 (0.42 - 1.3) | 0.30 |  |
|  | Category 4 | 67 (17.6) | 74 (18.2) | 0.81 (0.47 - 1.38) | 0.43 |  |
|  | Category 5 | 21 (5.5) | 19 (4.7) | 0.99 (0.47 - 2.09) | 0.97 |  |
|  | crossbreed | 82 (21.6) | 95 (23.4) | 0.77 (0.46 - 1.29) | 0.32 |  |
|  | No category | 78 (20.5) | 69 (17.0) | 1.01 (0.59 - 1.71) | 0.98 |  |
| Max. tumour size (cm, quartiles) | 0.2-3.5 | 22 (5.8) | 42 (10.3) | 1 (1 - 1) |  | 0.47 |
|  | 10.0-23.0 | 11 (2.9) | 15 (3.7) | 1.4 (0.55 - 3.56) | 0.48 |  |
|  | 3.5-6.0 | 27 (7.1) | 39 (9.6) | 1.32 (0.65 - 2.69) | 0.44 |  |
|  | 6.0-10.0 | 30 (7.9) | 52 (12.8) | 1.1 (0.56 - 2.18) | 0.78 |  |
|  | no measurement available | 290 (76.3) | 258 (63.5) | 2.15 (1.25 - 3.69) | <0.01 |  |
| Max. tumour size (cm, terciles) | 0.2-4.1 | 30 (7.9) | 49 (12.1) | 1 (1 - 1) |  | 0.88 |
|  | 4.1-8.0 | 34 (8.9) | 53 (13.1) | 1.05 (0.56 - 1.96) | 0.88 |  |
|  | 8.0-23.0 | 26 (6.8) | 46 (11.3) | 0.92 (0.48 - 1.79) | 0.81 |  |
|  | no measurement available | 290 (76.3) | 258 (63.5) | 1.84 (1.13 - 2.98) | 0.01 |  |
