# Supplementary material for: Survival time and prognostic factors in dogs clinically diagnosed with haemangiosarcoma in UK first opinion practice
Source: PLoS One. 2025 Jun 6;20(6):e0316066. doi: 10.1371/journal.pone.0316066 (PMC12143555; doi:10.1371/journal.pone.0316066)
Supplement: S3 Table — Percentages shown are column percentages. (DOCX) [file pone.0316066.s003.docx]

# Supplementary material - Survival time and prognostic factors in dogs clinically diagnosed with haemangiosarcoma in UK first opinion practice

**Table S3 -** Descriptive statistics and univariable Cox proportional hazards regression results of risk factors evaluated for hazard of time to death in haemangiosarcoma cases diagnosed in first-opinion practices in VetCompass in 2019 with ≥1 day of survival post presentation (n=407). Percentages shown are column percentages.

| variable |  | no. of cases (%) | HR (95% CI) | p_value | LRT p-value |
| --- | --- | --- | --- | --- | --- |
| Corporate group | Group A | 80 (19.7) | 1 | 1 | 0.04 |
|  | Group B | 158 (38.9) | 1.31 (0.96-1.78) | 0.10 |  |
|  | Group C | 7 (1.7) | 0.54 (0.20-1.50) | 0.24 |  |
|  | Group D | 111 (27.3) | 1.43 (1.03-2.00) | 0.03 |  |
|  | Group E | 50 (12.3) | 1.53 (1.04-2.27) | 0.03 |  |
| Sex | female | 183 (45.1) | 1 | 1 | 0.02 |
|  | male | 220 (54.2) | 1.32 (1.06-1.64) | 0.01 |  |
|  | unrecorded | 3 (0.7) | 2.65 (0.84-8.35) | 0.1 |  |
| Sex-neuter status | female entire | 33 (8.1) | 1 | 1 | 0.01 |
|  | female neutered | 150 (36.9) | 0.55 (0.36-0.82) | 0.01 |  |
|  | male entire | 72 (17.7) | 0.76 (0.49-1.19) | 0.23 |  |
|  | male neutered | 148 (36.5) | 0.80 (0.54-1.21) | 0.29 |  |
|  | unrecorded entire | 2 (0.5) | 2.76 (0.65-11.67) | 0.17 |  |
|  | unrecorded neutered | 1 (0.2) | 0.87 (0.12-6.40) | 0.89 |  |
| Neuter | entire | 107 (26.4) | 1 | 1 | 0.04 |
|  | neutered | 299 (73.6) | 0.78 (0.61-0.99) | 0.04 |  |
| Age at diagnosis (quartiles, years) | 5-7 | 19 (4.7) | 1 | 1 | 0.01 |
|  | 7-9 | 87 (21.4) | 1.58 (0.87-2.86) | 0.13 |  |
|  | 9-11 | 144 (35.5) | 2.31 (1.29-4.11) | 0.05 |  |
|  | 11-13 | 106 (26.1) | 2.31 (1.29-4.16) | 0.01 |  |
|  | 13-15 | 46 (11.3) | 2.29 (1.22-4.28) | 0.01 |  |
|  | Above 15 | 4 (1.0) | 1.78 (0.58-5.48) | 0.31 |  |
| Age at diagnosis (continuous) | Mean (SD) | 10.4 (2.1) | 1.08 (1.03-1.13) | <0.01 | <0.01 |
| Patient neutering status prior to presentation | Neutered prior to presentation | 301 (74.1) | 1 | 1 | 0.01 |
|  | Entire | 102 (25.1) | 1.37 (1.07-1.74) | 0.01 |  |
|  | Neutered after presentation | 3 (0.7) | 0.24 (0.03-1.74) | 0.16 |  |
| Age at neutering (years) | Mean (SD) | 4.1 (3.6) | 1.01 (0.93-1.09) | 0.81 | 0.81 |
| Presumptive diagnosis | Definitive | 297 (73.2) | 1 | 1 | <0.001 |
|  | Presumptive only | 109 (26.8) | 1.63 (1.28-2.07) | <0.001 |  |
| Time between neutering and diagnosis (continuous, years) | Mean (SD) | 5.4 (2.9) | 1.05 (0.94-1.16) | 0.39 | 0.38 |
| Maximum tumour size (continuous, cm) | Mean (SD) | 6.5 (4.2) | 1.07 (1.03-1.11) | <0.001 | <0.001 |
| Haematological clinical signs present | No haematological signs | 291 (71.7) | 1 | 1 | <0.001 |
|  | Haematological signs present | 115 (28.3) | 1.62 (1.28-2.05) | <0.001 |  |
| Cardiac clinical signs present | No cardiac signs | 345 (85.0) | 1 | 1 | 0.20 |
|  | Cardiac signs present | 61 (15.0) | 1.22 (0.90-1.65) | 0.19 |  |
| Respiratory clinical signs present | No respiratory signs | 374 (92.1) | 1 | 1 | 0.810 |
|  | Respiratory signs present | 32 (7.9) | 1.05 (0.72-1.53) | 0.81 |  |
| Gastrointestinal clinical signs present | No gastrointestinal signs | 347 (85.5) | 1 | 1 | 0.092 |
|  | Gastrointestinal signs present | 59 (14.5) | 1.31 (0.97-1.77) | 0.082 |  |
| Urinary clinical signs present | No Urinary signs | 373 (91.9) | 1 | 1 | 0.22 |
|  | Urinary signs present | 33 (8.1) | 1.28 (0.87-1.87) | 0.21 |  |
| Non-specific clinical signs present | No non-specific signs | 147 (36.2) | 1 | 1 | <0.001 |
|  | Non-specific signs present | 259 (63.8) | 1.80 (1.43-2.27) | <0.001 |  |
| Other clinical signs present | No other signs | 147 (36.2) | 1 | 1 | <0.001 |
|  | Other signs present | 259 (63.8) | 1.80 (1.43-2.27) | <0.001 |  |
| Mass associated clinical signs present | No mass associated signs | 220 (54.2) | 1 | 1 | <0.001 |
|  | Mass associated signs present | 186 (45.8) | 0.60 (0.48-0.75) | <0.001 |  |
| No clinical signs present | Clinical signs present | 380 (93.6) | 1 | 1 | 0.131 |
|  | No clinical signs present | 26 (6.4) | 1.44 (0.92-2.27) | 0.112 |  |
| Imaging performed | No imaging performed | 95 (23.4) | 1 | 1 | <0.001 |
|  | Imaging performed | 311 (76.6) | 1.74 (1.33-2.28) | <0.001 |  |
| Samples taken | No samples taken | 102 (25.1) | 1 | 1 | <0.01 |
|  | Samples taken | 304 (74.9) | 0.69 (0.54-0.88) | <0.01 |  |
| Laboratory tests performed | No lab tests performed | 131 (32.3) | 1 | 1 | 0.37 |
|  | Lab tests performed | 275 (67.7) | 1.11 (0.88-1.40) | 0.37 |  |
| Cardiac diagnostics performed | No cardiac diagnostics | 367 (90.4) | 1 | 1 | 0.53 |
|  | Cardiac diagnostics performed | 39 (9.6) | 1.12 (0.78-1.60) | 0.53 |  |
| Abdominal diagnostics performed | No Abdominal diagnostics | 143 (35.2) | 1 | 1 | <0.001 |
|  | Abdominal diagnostics performed | 263 (64.8) | 1.80 (1.42-2.27) | <0.001 |  |
| No diagnostics performed | Diagnostics performed | 398 (98.0) | 1 | 1 | 0.58 |
|  | No diagnostics performed | 8 (2.0) | 0.76 (0.28-2.05) | 0.59 |  |
| Any surgical management performed | No surgery | 126 (31.0) | 1 | 1 | <0.001 |
|  | Surgery | 280 (69.0) | 0.46 (0.37-0.59,) | <0.001 |  |
| Any medical management performed | No medicine | 268 (66.0) | 1 | 1 | 0.56 |
|  | Medicine | 138 (34.0) | 1.07 (0.85-1.34) | 0.56 |  |
| Any cardiac medical management performed | No cardiac medicine | 403 (99.3) | 1 | 1 | 0.78 |
|  | Cardiac medicine | 3 (0.7) | 0.86 (0.27-2.67) | 0.79 |  |
| Any alter1tive medical management performed | No alter1tive medicine | 397 (97.8) | 1 | 1 | 0.69 |
|  | Alter1tive medicine | 9 (2.2) | 1.15 (0.59-2.23) | 0.68 |  |
| Any transfusion medical management performed | No transfusion | 380 (93.6) | 1 | 1 | 0.05 |
|  | Transfusion | 26 (6.4) | 1.56 (1.03-2.37) | 0.04 |  |
| Any haemostatic medical management performed | No haemostatic medicine | 372 (91.6) | 1 | 1 | 0.03 |
|  | Haemostatic medicine | 34 (8.4) | 1.54 (1.06-2.23) | 0.02 |  |
| Any palliative medical management performed | No palliative medicine | 327 (80.5) | 1 | 1 | 0.05 |
|  | Palliative medicine | 79 (19.5) | 1.31 (1.01-1.70) | 0.05 |  |
| No medical management performed | Medicine | 138 (34.0) | 1 | 1 | 0.56 |
|  | No medicine | 268 (66.0) | 0.94 (0.75-1.17) | 0.56 |  |
| No surgical management performed | Surgery | 280 (69.0) | 1 | 1 | <0.001 |
|  | No surgery | 126 (31.0) | 2.15 (1.70-2.72) | <0.001 |  |
| Medical and surgical management performed | No medicine and surgery | 317 (78.1) | 1 | 1 | 0.08 |
|  | Medicine and surgery | 89 (21.9) | 0.80 (0.62-1.03) | 0.09 |  |
| No medical or surgical management performed | Medicine or surgery | 329 (81.0) | 1 | 1 | <0.001 |
|  | No medicine or surgery | 77 (19.0) | 1.89 (1.45-2.47,) | <0.001 |  |
| Visited a referral centre | No referral centre | 328 (80.8) | 1 | 1 | <0.01 |
|  | Visited referral centre | 78 (19.2) | 0.66 (0.49-0.88) | <0.01 |  |
| Abdominal metastases present | No Abdominal metastases | 349 (86.0) | 1 | 1 | 0.24 |
|  | Abdominal metastases | 57 (14.0) | 1.21 (0.89-1.65) | 0.23 |  |
| Thoracic metastases present | No thoracic metastases | 384 (94.6) | 1 | 1 | 0.09 |
|  | Thoracic metastases | 22 (5.4) | 1.51 (0.97-2.35) | 0.07 |  |
| Cranial metastases present | No cranial metastases | 404 (99.5) | 1 | 1 | 0.71 |
|  | Cranial metastases | 2 (0.5) | 1.32 (0.33-5.30) | 0.70 |  |
| Soft tissue metastases present | No soft tissue metastases | 390 (96.1) | 1 | 1 | 0.01 |
|  | Soft tissue metastases | 16 (3.9) | 0.44 (0.24-0.81) | 0.01 |  |
| Lymph node metastases present | No lymph node metastases | 402 (99.0) | 1 | 1 | 0.13 |
|  | Lymph node metastases | 4 (1.0) | 2.38 (0.88-6.39) | 0.09 |  |
| Unspecified metastases present | No unspecified metastases | 401 (98.8) | 1 | 1 | 0.07 |
|  | Unspecified metastases | 5 (1.2) | 2.55 (1.05-6.18) | 0.04 |  |
| Any metastases present | No metastases present | 316 (77.8) | 1 | 1 | 0.61 |
|  | Metastases present | 90 (22.2) | 1.07 (0.83-1.38) | 0.61 |  |
| Cardiac interest location | Cardiac | 17 (4.2) | 1 | 1 | <0.001 |
|  | No location specified | 249 (61.3) | 0.70 (0.42-1.14) | 0.15 |  |
|  | Non cardiac cutaneous | 124 (30.5) | 0.32 (0.19-0.54) | <0.001 |  |
|  | Non cardiac visceral | 16 (3.9) | 0.84 (0.41-1.71) | 0.65 |  |
| Splenic interest location | No location specified | 221 (54.4) | 1 | 1 | <0.001 |
|  | Non splenic cutaneous | 45 (11.1) | 1.16 (0.83-1.63) | 0.39 |  |
|  | Non splenic visceral | 124 (30.5) | 0.46 (0.36-0.59) | <0.001 |  |
|  | Splenic | 16 (3.9) | 1.21 (0.70-2.09) | 0.487 |  |
| Hepatic interest location | Hepatic | 43 (10.6) | 1 | 1 | <0.001 |
|  | No location specified | 223 (54.9) | 0.84 (0.60-1.18) | 0.31 |  |
|  | Non hepatic cutaneous | 124 (30.5) | 0.39 (0.27-0.56) | <0.001 |  |
|  | Non hepatic visceral | 16 (3.9) | 1.02 (0.55-1.88) | 0.94 |  |
| Clinic postcode urban-rural status | Mixed urban/rural | 193 (51.2) | 1 | 1 | 0.77 |
|  | Rural | 63 (16.7) | 1.07 (0.78-1.47) | 0.69 |  |
|  | Urban | 121 (32.1) | 1.09 (0.85-1.40) | 0.49 |  |
| Clinic postcode IMD quintile | 1 (most deprived) | 48 (12.7) | 1 | 1 | 0.44 |
|  | 2 | 82 (21.8) | 0.98 (0.66-1.46) | 0.92 |  |
|  | 3 | 78 (20.7) | 1.22 (0.82-1.82) | 0.32 |  |
|  | 4 | 85 (22.5) | 1.24 (0.84-1.83) | 0.28 |  |
|  | 5 (least deprived) | 84 (22.3) | 0.99 (0.66-1.47) | 0.96 |  |
| Top 20 VetCompass breeds | Crossbreed | 95 (23.4) | 1 | 1 | 0.10 |
|  | Bichon Frise | 5 (1.2) | 2.80 (1.01-7.73) | 0.05 |  |
|  | Border Collie | 13 (3.2) | 1.04 (0.55-1.96) | 0.91 |  |
|  | Border Terrier | 1 (0.2) | 0.00 (0.00-Inf) | 0.99 |  |
|  | Cavalier King Charles Spaniel | 4 (1.0) | 1.40 (0.44-4.44) | 0.57 |  |
|  | Cockapoo | 1 (0.2) | 13.83 (1.87-102.42) | 0.01 |  |
|  | English Cocker Spaniel | 17 (4.2) | 1.38 (0.79-2.41) | 0.25 |  |
|  | English Springer Spaniel | 9 (2.2) | 0.73 (0.29-1.80) | 0.49 |  |
|  | French Bulldog | 1 (0.2) | 3.71 (0.51-26.86) | 0.20 |  |
|  | German Shepherd Dog | 41 (10.1) | 1.56 (1.04-2.35) | 0.03 |  |
|  | Golden Retriever | 16 (3.9) | 0.86 (0.44-1.68) | 0.66 |  |
|  | Jack Russell Terrier | 12 (3.0) | 0.85 (0.44-1.64) | 0.62 |  |
|  | Labrador Retriever | 48 (11.8) | 1.05 (0.73-1.52) | 0.79 |  |
|  | Miniature Dachshund | 2 (0.5) | 0.43 (0.11-1.77) | 0.24 |  |
|  | Staffordshire Bull Terrier | 19 (4.7) | 0.84 (0.50-1.43) | 0.52 |  |
|  | West Highland White Terrier | 8 (2.0) | 1.70 (0.78-3.69) | 0.18 |  |
|  | Yorkshire Terrier | 2 (0.5) | 0.50 (0.07-3.57) | 0.49 |  |
|  | Other breed | 112 (27.6) | 0.85 (0.63-1.16) | 0.31 |  |
| Breeds with >=5 cases | Crossbreed | 95 (23.4) | 1 | 1 | 0.18 |
|  | Beagle | 12 (3.0) | 0.65 (0.32-1.29) | 0.21 |  |
|  | Bichon Frise | 5 (1.2) | 2.82 (1.02-7.79) | 0.05 |  |
|  | Border Collie | 13 (3.2) | 1.04 (0.55-1.97) | 0.90 |  |
|  | Boxer | 15 (3.7) | 1.08 (0.59-1.99) | 0.81 |  |
|  | Cavalier King Charles Spaniel | 4 (1.0) | 1.41 (0.44-4.48) | 0.56 |  |
|  | Dogue de Bordeaux | 1 (0.2) | 2.62 (0.36-18.94) | 0.34 |  |
|  | English Cocker Spaniel | 17 (4.2) | 1.39 (0.80-2.42) | 0.24 |  |
|  | English Springer Spaniel | 9 (2.2) | 0.73 (0.29-1.80) | 0.49 |  |
|  | Flat Coated Retriever | 6 (1.5) | 2.85 (1.23-6.58) | 0.01 |  |
|  | German Shepherd Dog | 41 (10.1) | 1.57 (1.05-2.37) | 0.03 |  |
|  | Golden Retriever | 16 (3.9) | 0.86 (0.44-1.67) | 0.65 |  |
|  | Hungarian Vizsla | 8 (2.0) | 1.21 (0.56-2.63) | 0.63 |  |
|  | Jack Russell Terrier | 12 (3.0) | 0.85 (0.44-1.64) | 0.63 |  |
|  | Labradoodle | 5 (1.2) | 0.84 (0.31-2.29) | 0.73 |  |
|  | Labrador Retriever | 48 (11.8) | 1.05 (0.73-1.52) | 0.78 |  |
|  | Lurcher | 5 (1.2) | 0.79 (0.29-2.16) | 0.64 |  |
|  | Miniature Sch1uzer | 8 (2.0) | 0.73 (0.34-1.59) | 0.43 |  |
|  | Rottweiler | 2 (0.5) | 0.50 (0.07-3.59) | 0.49 |  |
|  | Staffordshire Bull Terrier | 19 (4.7) | 0.84 (0.50-1.43) | 0.53 |  |
|  | West Highland White Terrier | 8 (2.0) | 1.70 (0.78-3.70) | 0.18 |  |
|  | Other breed | 57 (14.0) | 0.76 (0.53-1.09) | 0.13 |  |
| Breeds with >=10 cases | Crossbreed | 95 (23.4) | 1 | 1 | 0.42 |
|  | Beagle | 12 (3.0) | 0.65 (0.32-1.29) | 0.22 |  |
|  | Bichon Frise | 5 (1.2) | 2.76 (1.00-7.63) | 0.05 |  |
|  | Border Collie | 13 (3.2) | 1.04 (0.55-1.96) | 0.91 |  |
|  | Boxer | 15 (3.7) | 1.08 (0.59-1.99) | 0.81 |  |
|  | English Cocker Spaniel | 17 (4.2) | 1.38 (0.79-2.41) | 0.26 |  |
|  | English Springer Spaniel | 9 (2.2) | 0.73 (0.29-1.80) | 0.49 |  |
|  | German Shepherd Dog | 41 (10.1) | 1.56 (1.04-2.34) | 0.03 |  |
|  | Golden Retriever | 16 (3.9) | 0.86 (0.44-1.68) | 0.66 |  |
|  | Hungarian Vizsla | 8 (2.0) | 1.20 (0.55-2.61) | 0.65 |  |
|  | Jack Russell Terrier | 12 (3.0) | 0.85 (0.44-1.64) | 0.62 |  |
|  | Labradoodle | 5 (1.2) | 0.84 (0.31-2.29) | 0.73 |  |
|  | Labrador Retriever | 48 (11.8) | 1.05 (0.73-1.52) | 0.79 |  |
|  | Lurcher | 5 (1.2) | 0.79 (0.29-2.16) | 0.64 |  |
|  | Miniature Sch1uzer | 8 (2.0) | 0.73 (0.34-1.59) | 0.43 |  |
|  | Rottweiler | 2 (0.5) | 0.50 (0.07-3.59) | 0.49 |  |
|  | Staffordshire Bull Terrier | 19 (4.7) | 0.84 (0.50-1.43) | 0.52 |  |
|  | West Highland White Terrier | 8 (2.0) | 1.69 (0.78-3.67) | 0.18 |  |
|  | other_breed | 68 (16.7) | 0.86 (0.61-1.21) | 0.37 |  |
| Ancestral group breed | Ancient group | 3 (0.7) | 1 | 1 | 0.81 |
|  | Crossbreed | 95 (23.4) | 0.91 (0.29-2.90) | 0.88 |  |
|  | Herding sighthound group | 19 (4.7) | 0.87 (0.25-2.96) | 0.82 |  |
|  | Mastiff terrier group | 129 (31.8) | 0.93 (0.30-2.93) | 0.90 |  |
|  | Modern group | 92 (22.7) | 0.92 (0.29-2.92) | 0.89 |  |
|  | Mountain group | 4 (1.0) | 0.33 (0.06-2.00) | 0.23 |  |
|  | No ancestral group | 64 (15.8) | 0.89 (0.28-2.85) | 0.86 |  |
| Genotype group breed | Category 1 | 95 (23.4) | 1 | 1 | 0.26 |
|  | Category 2 | 41 (10.1) | 1.54 (1.02-2.31) | 0.04 |  |
|  | Category 3 | 49 (12.1) | 0.80 (0.54-1.19) | 0.28 |  |
|  | Category 4 | 59 (14.5) | 0.96 (0.67-1.38) | 0.82 |  |
|  | Category 5 | 74 (18.2) | 1.02 (0.73-1.42) | 0.92 |  |
|  | crossbreed | 19 (4.7) | 0.95 (0.56-1.61) | 0.86 |  |
|  | No category | 69 (17.0) | 0.95 (0.67-1.33) | 0.75 |  |
| Max. tumour size (cm, quartiles) | 0.2-3.5 | 42 (10.3) | 1 | 1 | <0.001 |
|  | 10.0-23.0 | 39 (9.6) | 2.17 (1.28-3.66) | 0.004 |  |
|  | 3.5-6.0 | 52 (12.8) | 2.81 (1.71-4.62) | <0.001 |  |
|  | 6.0-10.0 | 15 (3.7) | 2.80 (1.45-5.43) | 0.002 |  |
|  | no measurement available | 258 (63.5) | 2.86 (1.89-4.34) | <0.001 |  |
| Chemotherapy dose | High dose chemotherapy | 27 (6.7) | 1 | 1 | 0.03 |
|  | Metronomic chemotherapy | 10 (2.5) | 1.53 (0.69-3.36) | 0.30 |  |
|  | No chemotherapy | 369 (90.9) | 1.76 (1.12-2.77) | 0.02 |  |
| Chemotherapy - doxi/epirubicin or other | Doxi/epirubicin chemotherapy | 26 (6.4) | 1 | 1 | 0.031 |
|  | No chemotherapy recorded | 379 (93.3) | 1.75 (1.10-2.78) | 0.03 |  |
|  | Not doxi/epirubicin chemotherapy | 1 (0.2) | 1.00 (0.13-7.45) | 0.99 |  |
| Any haemangiosarcoma cardiac location | No cutaneous location | 389 (95.8) | 1 | 1 | 0.02 |
|  | Cutaneous location | 17 (4.2) | 1.87 (1.14-3.05) | 0.01 |  |
| Any haemangiosarcoma splenic location | No hepatic location | 177 (43.6) | 1 | 1 | <0.001 |
|  | Hepatic location | 229 (56.4) | 1.62 (1.30-2.02) | <0.001 |  |
| Any haemangiosarcoma hepatic location | No splenic location | 331 (81.5) | 1 | 1 | <0.01 |
|  | Splenic location | 75 (18.5) | 1.52 (1.16-1.99) | <0.01 |  |
| Any haemangiosarcoma cutaneous location | No cardiac location | 268 (66.0) | 1 | 1 | <0.001 |
|  | Cardiac location | 138 (34.0) | 0.49 (0.39-0.63) | <0.001 |  |
| Max tumour size (cm, terciles) | 0.2-4.1 | 49 (12.1) | 1 | 1 | <0.001 |
|  | 4.1-8.0 | 53 (13.1) | 2.07 (1.30-3.28) | <0.01 |  |
|  | 8.0-23.0 | 46 (11.3) | 2.48 (1.55-3.98) | <0.001 |  |
|  | no measurement available | 258 (63.5) | 2.51 (1.73-3.65) | <0.001 |  |
