# Supplementary material for: Survival time and prognostic factors in dogs clinically diagnosed with haemangiosarcoma in UK first opinion practice
Source: PLoS One. 2025 Jun 6;20(6):e0316066. doi: 10.1371/journal.pone.0316066 (PMC12143555; doi:10.1371/journal.pone.0316066)
Supplement: S4 Table — Percentages shown are column percentages. (DOCX) [file pone.0316066.s004.docx]

# Supplementary material - Survival time and prognostic factors in dogs clinically diagnosed with haemangiosarcoma in UK first opinion practice

**Table S4 -** Descriptive statistics and univariable Cox proportional hazards regression results of risk factors evaluated for hazard of time to death in haemangiosarcoma cases diagnosed with histopathology in first-opinion practices in VetCompass in 2019 (n=272). Percentages shown are column percentages.

| Variable |  | no. of cases (%) | HR (95% CI) | p-value | LRT p-value |
| --- | --- | --- | --- | --- | --- |
| Corporate group | Group A | 40 (19.2) | 1 | 1 | 0.119 |
|  | Group B | 84 (40.4) | 1.47 (1.01-2.15) | 0.044 |  |
|  | Group C | 3 (1.4) | 0.71 (0.22-2.29) | 0.6 |  |
|  | Group D | 50 (24.0) | 1.30 (0.86-1.98) | 0.2 |  |
|  | Group E | 31 (14.9) | 1.67 (1.04-2.67) | 0.034 |  |
| Sex | Female | 101 (48.6) | 1 | 1 | 0.151 |
|  | Male | 105 (50.5) | 1.26 (0.96-1.66) | 0.095 |  |
|  | Unrecorded | 2 (1.0) | 2.50 (0.61-10.2) | 0.2 |  |
| Sex-neuter status | Female entire | 17 (8.2) | 1 | 1 | 0.115 |
|  | Female neutered | 84 (40.4) | 0.57 (0.34-0.96) | 0.035 |  |
|  | Male entire | 41 (19.7) | 0.85 (0.48-1.50) | 0.6 |  |
|  | Male neutered | 64 (30.8) | 0.73 (0.43-1.26) | 0.3 |  |
|  | Unrecorded entire | 1 (0.5) | 3.24 (0.43-24.70) | 0.3 |  |
|  | Unrecorded neutered | 1 (0.5) | 1.02 (0.13-7.66) | >0.9 |  |
| Neuter | Entire | 59 (28.4) | 1 | 1 | 0.026 |
|  | Neutered | 149 (71.6) | 0.70 (0.52-0.95) | 0.022 |  |
| Age at diagnosis (quartiles, years) | 5-7 | 11 (5.3) | 1 | 1 | 0.051 |
|  | >7-9 | 46 (22.1) | 1.27 (0.65-2.45) | 0.5 |  |
|  | >9-11 | 80 (38.5) | 1.97 (1.04-3.71) | 0.037 |  |
|  | >11-13 | 46 (22.1) | 2.01 (1.04-3.89) | 0.039 |  |
|  | >13-15 | 23 (11.1) | 1.97 (0.95-4.07) | 0.066 |  |
|  | Above 15 | 2 (1.0) | 1.22 (0.27-5.52) | 0.8 |  |
| Age at diagnosis continuous | Mean (SD) | 10.09 (2.06) | 1.09 (1.03-1.16) | 0.005 | 0.006 |
| Patient neutering status prior to presentation | Neutered prior to presentation | 151 (72.6) | 1 | 1 | 0.005 |
|  | Entire | 56 (26.9) | 1.56 (1.14-2.12) | 0.005 |  |
|  | Neutered after presentation | 1 (0.5) | 0.27 (0.04-1.97) | 0.2 |  |
| Time between neutering and diagnosis (continuous, years) | Mean (SD) | 5.04(2.94) | 1.07 | 0.2 | 0.203 |
| Age at neutering (years) | Mean (SD) | 4.44(3.68) | 1.07 | 0.2 | 0.203 |
| Maximum tumour size (continuous, cm) | Mean (SD) | 6.25(4.58) | 1.08 | <0.001 | <0.001 |
| Haematological clinical signs present | No haematological signs | 150 (72.1) | 1 | 1 | <0.001 |
|  | Haematological signs present | 58 (27.9) | 1.72 (1.27-2.34) | <0.001 |  |
| Cardiac clinical signs present | No cardiac signs | 185 (88.9) | 1 | 1 | 0.231 |
|  | Cardiac signs present | 23 (11.1) | 1.32 (0.85-2.03) | 0.2 |  |
| Respiratory clinical signs present | No respiratory signs | 194 (93.3) | 1 | 1 | 0.674 |
|  | Respiratory signs present | 14 (6.7) | 0.89 (0.52-1.54) | 0.7 |  |
| Gastrointestinal clinical signs present | No gastrointestinal signs | 185 (88.9) | 1 | 1 | 0.145 |
|  | Gastrointestinal signs present | 23 (11.1) | 1.40 (0.91-2.17) | 0.13 |  |
| Urinary clinical signs present | No urinary signs | 194 (93.3) | 1 | 1 | 0.749 |
|  | Urinary signs present | 14 (6.7) | 0.92 (0.53-1.58) | 0.8 |  |
| Non-specific clinical signs present | No non-specific signs | 91 (43.8) | 1 | 1 | <0.001 |
|  | Non-specific signs present | 117 (56.2) | 1.65 (1.25-2.17) | <0.001 |  |
| Other clinical signs present | No other signs | 91 (43.8) | 1 | 1 | <0.001 |
|  | Other signs present | 117 (56.2) | 1.65 (1.25-2.17) | <0.001 |  |
| Mass associated clinical signs present | No mass associated signs | 102 (49.0) | 1 | 1 | <0.001 |
|  | Mass associated signs present | 106 (51.0) | 0.60 (0.46-0.79) | <0.001 |  |
| No clinical signs present | Clinical signs present | 194 (93.3) | 1 | 1 | 0.129 |
|  | No clinical signs present | 14 (6.7) | 1.57 (0.91-2.70) | 0.11 |  |
| Imaging performed | No imaging performed | 57 (27.4) | 1 | 1 | 0.003 |
|  | Imaging performed | 151 (72.6) | 1.57 (1.15-2.13) | 0.004 |  |
| Cardiac diagnostics performed | No cardiac diagnostics | 190 (91.3) | 1 | 1 | 0.842 |
|  | Cardiac diagnostics performed | 18 (8.7) | 0.95 (0.59-1.55) | 0.8 |  |
| Abdominal diagnostics performed | No Abdominal diagnostics | 82 (39.4) | 1 | 1 | <0.001 |
|  | Abdominal diagnostics performed | 126 (60.6) | 1.67 (1.26-2.20) | <0.001 |  |
| Any surgical management performed | No surgery | 29 (13.9) | 1 | 1 | 0.001 |
|  | Surgery | 179 (86.1) | 0.48 (0.32-0.72) | <0.001 |  |
| Any medical management performed | No medicine | 138 (66.3) | 1 | 1 | 0.972 |
|  | Medicine | 70 (33.7) | 1.01 (0.75-1.34) | >0.9 |  |
| Any cardiac medical management performed | No cardiac medicine | 207 (99.5) | 1 | 1 | 0.854 |
|  | Cardiac medicine | 1 (0.5) | 0.84 (0.12-5.98) | 0.9 |  |
| Any alter1tive medical management performed | No alter1tive medicine | 203 (97.6) | 1 | 1 | 0.666 |
|  | Alter1tive medicine | 5 (2.4) | 1.22 (0.50-2.99) | 0.7 |  |
| Any transfusion medical management performed | No transfusion | 190 (91.3) | 1 | 1 | 0.017 |
|  | Transfusion | 18 (8.7) | 1.91 (1.18-3.12) | 0.009 |  |
| Any haemostatic medical management performed | No haemostatic medicine | 187 (89.9) | 1 | 1 | 0.021 |
|  | Haemostatic medicine | 21 (10.1) | 1.78 (1.13-2.81) | 0.013 |  |
| Any palliative medical management performed | No palliative medicine | 174 (83.7) | 1 | 1 | 0.804 |
|  | Palliative medicine | 34 (16.3) | 1.05 (0.72-1.51) | 0.8 |  |
| No medical management performed | Medicine | 70 (33.7) | 1 | 1 | 0.972 |
|  | No medicine | 138 (66.3) | 1.0 (0.74-1.33) | >0.9 |  |
| No surgical management performed | Surgery | 179 (86.1) | 1 | 1 | 0.001 |
|  | No surgery | 29 (13.9) | 2.07 (1.39-3.08) | <0.001 |  |
| Medical and surgical management performed | No medicine and surgery | 148 (71.2) | 1 | 1 | 0.756 |
|  | Medicine and surgery | 60 (28.8) | 0.95 (0.71-1.29) | 0.8 |  |
| No medical or surgical management performed | Medicine or surgery | 189 (90.9) | 1 | 1 | <0.001 |
|  | No medicine or surgery | 19 (9.1) | 2.69 (1.67-4.35) | <0.001 |  |
| Visited a referral centre | No referral centre | 175 (84.1) | 1 | 1 | <0.001 |
|  | Visited referral centre | 33 (15.9) | 0.49 (0.34-0.72) | <0.001 |  |
| Abdominal metastases present | No Abdominal metastases | 176 (84.6) | 1 | 1 | 0.090 |
|  | Abdominal metastases | 32 (15.4) | 1.41 (0.96-2.07) | 0.078 |  |
| Thoracic metastases present | No thoracic metastases | 195 (93.8) | 1 | 1 | 0.232 |
|  | Thoracic metastases | 13 (6.2) | 1.44 (0.82-2.53) | 0.2 |  |
| Cranial metastases present | No cranial metastases | 206 (99.0) | 1 | 1 | 0.550 |
|  | Cranial metastases | 2 (1.0) | 1.58 (0.39-6.38) | 0.5 |  |
| Soft tissue metastases present | No soft tissue metastases | 198 (95.2) | 1 | 1 | 0.012 |
|  | Soft tissue metastases | 10 (4.8) | 0.48 (0.25-0.91) | 0.024 |  |
| Lymph node metastases present | No lymph node metastases | 205 (98.6) | 1 | 1 | 0.014 |
|  | Lymph node metastases | 3 (1.4) | 6.49 (2.04-20.7) | 0.002 |  |
| Unspecified metastases present | No unspecified metastases | 207 (99.5) | 1 | 1 | 0.941 |
|  | Unspecified metastases | 1 (0.5) | 1.08 (0.15-7.71) | >0.9 |  |
| Any metastases present | No metastases present | 153 (73.6) | 1 | 1 | 0.314 |
|  | Metastases present | 55 (26.4) | 1.17 (0.86-1.60) | 0.3 |  |
| Cardiac interest location | Cardiac | 1 (0.5) | 1 | 1 | <0.001 |
|  | Non cardiac visceral | 122 (58.7) | 0.19 (0.03-1.36) | 0.10 |  |
|  | Non cardiac cutaneous | 82 (39.4) | 0.09 (0.01-0.63) | 0.016 |  |
|  | No location specified | 3 (1.4) | 0.10 (0.01-1.02) | 0.052 |  |
| Splenic interest location | Splenic | 109 (52.4) | 1 | 1 | <0.001 |
|  | Non splenic visceral | 14 (6.7) | 1.68 (0.96-2.95) | 0.067 |  |
|  | Non splenic cutaneous | 82 (39.4) | 0.48 (0.36-0.65) | <0.001 |  |
|  | No location specified | 3 (1.4) | 0.58 (0.18-1.83) | 0.4 |  |
| Hepatic interest location | Hepatic | 11 (5.3) | 1 | 1 | <0.001 |
|  | Non hepatic visceral | 112 (53.8) | 0.63 (0.34-1.17) | 0.14 |  |
|  | Non hepatic cutaneous | 82 (39.4) | 0.30 (0.16-0.56) | <0.001 |  |
|  | No location specified | 3 (1.4) | 0.36 (0.10-1.29) | 0.12 |  |
| Clinic postcode urban-rural status | Mixed urban/rural | 103 (54.2) | 1 | 1 | 0.945 |
|  | Rural | 27 (14.2) | 1.04 (0.68-1.59) | 0.9 |  |
|  | Urban | 60 (31.6) | 0.96 (0.70-1.33) | 0.8 |  |
| Clinic postcode IMD quintile | 1 (most deprived) | 25 (13.2) | 1 | 1 | 0.971 |
|  | 2 | 51 (26.8) | 1.13 (0.70-1.87) | 0.6 |  |
|  | 3 | 37 (19.5) | 1.13 (0.68-1.88) | 0.6 |  |
|  | 4 | 38 (20.0) | 1.17 (0.70-1.94) | 0.5 |  |
|  | 5 (least deprived) | 39 (20.5) | 1.19 (0.72-1.97) | 0.5 |  |
| Top 20 VetCompass breeds | Crossbreed | 47 (22.9) | 1 | 1 | 0.245 |
|  | Bichon Frise | 0 (0.0) | 1 | 1 |  |
|  | Border Collie | 8 (3.9) | 1.00 (0.47-2.13) | >0.9 |  |
|  | Border Terrier | 0 (0.0) | 0.00 (0-inf) | >0.9 |  |
|  | Cavalier King Charles Spaniel | 0 (0.0) | 1 | 1 |  |
|  | Cockapoo | 0 (0.0) | 1 | 1 |  |
|  | English Cocker Spaniel | 8 (3.9) | 1.35 (0.63-2.86) | 0.4 |  |
|  | English Springer Spaniel | 3 (1.5) | 0.62 (0.19-2.01) | 0.4 |  |
|  | French Bulldog | 1 (0.5) | 5.08 (0.69-37.4) | 0.11 |  |
|  | German Shepherd Dog | 22 (10.7) | 1.66 (0.99-2.77) | 0.054 |  |
|  | Golden Retriever | 4 (2.0) | 0.87 (0.31-2.44) | 0.8 |  |
|  | Jack Russell Terrier | 6 (2.9) | 0.82 (0.35-1.91) | 0.6 |  |
|  | Labrador Retriever | 32 (15.6) | 1.38 (0.88-2.17) | 0.2 |  |
|  | Miniature Dachshund | 1 (0.5) | 0.34 (0.05-2.46) | 0.3 |  |
|  | Staffordshire Bull Terrier | 6 (2.9) | 0.75 (0.32-1.75) | 0.5 |  |
|  | West Highland White Terrier | 3 (1.5) | 1.25 (0.39-4.03) | 0.7 |  |
|  | Yorkshire Terrier | 1 (0.5) | 1.42 (0.19-10.3) | 0.7 |  |
|  | Other breed | 63 (30.7) | 0.83 (0.57-1.21) | 0.3 |  |
| Breeds with >=5 cases | Crossbreed | 47 (22.6) | 1 | 1 | 0.027 |
|  | Beagle | 7 (3.4) | 0.61 (0.27-1.35) | 0.2 |  |
|  | Bichon Frise | 3 (1.4) | 4.63 (1.40-15.4) | 0.012 |  |
|  | Border Collie | 8 (3.8) | 1.01 (0.48-2.15) | >0.9 |  |
|  | Boxer | 8 (3.8) | 1.19 (0.57-2.52) | 0.7 |  |
|  | Cavalier King Charles Spaniel | 0 (0.0) | 1 | 1 |  |
|  | Dogue de Bordeaux | 0 (0.0) | 1 | 1 |  |
|  | English Cocker Spaniel | 8 (3.8) | 1.36 (1.64-2.89) | 0.4 |  |
|  | English Springer Spaniel | 3 (1.4) | 0.62 (0.19-2.00) | 0.4 |  |
|  | Flat Coated Retriever | 6 (2.9) | 3.83 (1.62-9.06) | 0.002 |  |
|  | German Shepherd Dog | 22 (10.6) | 1.69 (1.01-2.83) | 0.046 |  |
|  | Golden Retriever | 4 (1.9) | 0.87 (0.31-2.42) | 0.8 |  |
|  | Hungarian Vizsla | 3 (1.4) | 2.51 (0.77-8.14) | 0.13 |  |
|  | Jack Russell Terrier | 6 (2.9) | 0.82 (0.35-1.92) | 0.6 |  |
|  | Labradoodle | 2 (1.0) | 0.51 (0.12-2.11) | 0.4 |  |
|  | Labrador Retriever | 32 (15.4) | 1.40 (0.89-2.20) | 0.15 |  |
|  | Lurcher | 4 (1.9) | 0.97 (0.35-2.69) | >0.9 |  |
|  | Miniature Schnauzer | 5 (2.4) | 0.91 (0.36-2.29) | 0.8 |  |
|  | Rottweiler | 0 (0.0) | 1 | 1 |  |
|  | Staffordshire Bull Terrier | 6 (2.9) | 0.74 (0.32-1.74) | 0.5 |  |
|  | West Highland White Terrier | 3 (1.4) | 1.26 (0.39-4.07) | 0.7 |  |
|  | Other breed | 31 (14.9) | 0.67 (0.42-1.05) | 0.081 |  |
| Breeds with >=10 cases | Crossbreed | 47 (22.6) | 1 | 1 | 0.221 |
|  | Beagle | 7 (3.4) | 0.61 (0.28-1.35) | 0.2 |  |
|  | Bichon Frise | 3 (1.4) | 4.47 (1.35-14.8) | 0.014 |  |
|  | Border Collie | 8 (3.8) | 1.00 (0.47-2.13) | >0.9 |  |
|  | Boxer | 8 (3.8) | 1.19 (0.56-2.52) | 0.7 |  |
|  | English Cocker Spaniel | 8 (3.8) | 1.35 (0.63-2.85) | 0.4 |  |
|  | English Springer Spaniel | 3 (1.4) | 0.62 (0.19-2.01) | 0.4 |  |
|  | German Shepherd Dog | 22 (10.6) | 1.66 (0.99-2.77) | 0.054 |  |
|  | Golden Retriever | 4 (1.9) | 0.87 (0.31-2.44) | 0.8 |  |
|  | Hungarian Vizsla | 3 (1.4) | 2.44 (0.75-7.90) | 0.14 |  |
|  | Jack Russell Terrier | 6 (2.9) | 0.82 (0.35-1.91) | 0.6 |  |
|  | Labradoodle | 2 (1.0) | 0.51 (0.12-2.10) | 0.4 |  |
|  | Labrador Retriever | 32 (15.4) | 1.38 (0.88-2.17) | 0.2 |  |
|  | Lurcher | 4 (1.9) | 0.96 (0.35-2.68) | >0.9 |  |
|  | Miniature Schnauzer | 5 (2.4) | 0.90 (0.36-2.28) | 0.8 |  |
|  | Rottweiler | 0 (0.0) | 1 | 1 |  |
|  | Staffordshire Bull Terrier | 6 (2.9) | 0.74 (0.32-1.75) | 0.5 |  |
|  | West Highland White Terrier | 3 (1.4) | 1.26 (0.39-4.04) | 0.7 |  |
|  | Other breed | 37 (17.8) | 0.78 (0.50-1.20) | 0.2 |  |
| Ancestral group breed | Ancient group | 1 (0.5) | 1 | 1 | 0.532 |
|  | Crossbreed | 47 (22.6) | 1.30 (0.18-9.48) | 0.8 |  |
|  | Herding sighthound group | 13 (6.2) | 1.29 (0.17-9.91) | 0.8 |  |
|  | Mastiff terrier group | 68 (32.7) | 1.59 (0.22-11.50) | 0.6 |  |
|  | Modern group | 45 (21.6) | 1.19 (0.16-8.64) | 0.9 |  |
|  | Mountain group | 1 (0.5) | 0.45 (0.03-7.25) | 0.6 |  |
|  | No ancestral group | 33 (15.9) | 1.16 (0.16-8.53) | 0.9 |  |
| Genotype group breed | Crossbreed | 47 (22.6) | 1 | 1 | 0.054 |
|  | Category 1 | 22 (10.6) | 1.63 (0.98-2.72) | 0.062 |  |
|  | Category 2 | 22 (10.6) | 0.66 (0.40-1.10) | 0.11 |  |
|  | Category 3 | 26 (12.5) | 1.01 (0.62-1.63) | >0.9 |  |
|  | Category 4 | 43 (20.7) | 1.33 (0.88-2.01) | 0.2 |  |
|  | Category 5 | 13 (6.2) | 1.00 (0.54-1.84) | >0.9 |  |
|  | No category | 35 (16.8) | 0.85 (0.55-1.32) | 0.5 |  |
| Max. tumour size (cm, quartiles) | 0.2-3.5 | 20 (9.6) | 1 | 1 | <0.001 |
|  | 10.0-23.0 | 23 (11.1) | 2.23 (1.21-4.11) | 0.010 |  |
|  | 3.5-6.0 | 27 (13.0) | 3.23 (1.79-5.82) | <0.001 |  |
|  | 6.0-10.0 | 11 (5.3) | 3.23 (1.52-6.85) | 0.002 |  |
|  | no measurement available | 127 (61.1) | 2.97 (1.83-4.81) | <0.001 |  |
| Max tumour size (cm, terciles) | 0.2-4.1 | 26 (12.5) | 1 | 1 | <0.001 |
|  | 4.1-8.0 | 27 (13.0) | 2.15 (1.24-3.73) | 0.007 |  |
|  | 8.0-23.0 | 28 (13.5) | 2.77 (1.60-4.76) | <0.001 |  |
|  | no measurement available | 127 (61.1) | 2.56 (1.66-3.94) | <0.001 |  |
| Chemotherapy dose | High dose chemotherapy | 17 (8.2) | 1 | 1 | 0.291 |
|  | Metronomic chemotherapy | 6 (2.9) | 1.42 (0.56-3.62) | 0.5 |  |
|  | No chemotherapy | 185 (88.9) | 1.46 (0.89-2.40) | 0.14 |  |
| Chemotherapy - doxi/epirubicin or other | Doxi/epirubicin chemotherapy | 16 (7.7) | 1 | 1 | 0.292 |
|  | No chemotherapy recorded | 191 (91.8) | 1.46 (0.87-2.43) | 0.15 |  |
|  | Not doxi/epirubicin chemotherapy | 1 (0.5) | 0.99 (0.13-7.45) | >0.9 |  |
| Any haemangiosarcoma cardiac location | No cutaneous location | 207 (99.5) | 1 | 1 | 0.138 |
|  | Cutaneous location | 1 (0.5) | 7.20 (0.98-52.7) | 0.052 |  |
| Any haemangiosarcoma splenic location | No hepatic location | 95 (45.7) | 1 | 1 | <0.001 |
|  | Hepatic location | 113 (54.3) | 1.75 (1.32-2.32) | <0.001 |  |
| Any haemangiosarcoma hepatic location | No splenic location | 177 (85.1) | 1 | 1 | 0.003 |
|  | Splenic location | 31 (14.9) | 1.88 (1.27-2.77) | 0.001 |  |
| Any haemangiosarcoma cutaneous location | No cutaneous location | 116 (55.8) | 1 | 1 | <0.001 |
|  | Cutaneous location | 92 (44.2) | 0.52 (0.40-0.70) | <0.001 |  |
