# Supplementary material for: Survival time and prognostic factors in dogs clinically diagnosed with haemangiosarcoma in UK first opinion practice
Source: PLoS One. 2025 Jun 6;20(6):e0316066. doi: 10.1371/journal.pone.0316066 (PMC12143555; doi:10.1371/journal.pone.0316066)
Supplement: Attachment — Submitted filename: 250414_letter to editor and reviewers PLOS ONE.docx [file pone.0316066.s006.docx]

Simon Clegg,

Academic Editor,

PLOS One

14th April 2025

RE: Manuscript ID: PONE-D-24-56041

Manuscript title: Survival time and prognostic factors in dogs diagnosed with haemangiosarcoma in UK first opinion practice

Updated manuscript title: Survival time and prognostic factors in dogs clinically diagnosed with haemangiosarcoma in UK first opinion practice

Dear Dr Clegg and reviewers,

Thank you for your advice on how best to revise this manuscript and for the helpful and constructive further feedback from the reviewers. We have addressed all comments from both reviewers, as outlined below, with implementation or justification of all. We are grateful for these suggestions and feel that they have substantially enhanced this paper. We hope that you agree that these changes ensure that the overall paper now meets the standard required for publication in PLOS One.

Reviewer comments appear in normal text followed by any author responses in italics and then the revised manuscript lines and wording in bold. The line numbers quoted refer to the tracked changes version of the re-submission. In the resubmitted manuscript changes are highlighted in yellow.

Once again, thank you to PLOS One and to your reviewers for your excellent advice and support.

Yours sincerely,

Collette Taylor

Reviewer #1: This manuscript is an interesting evaluation of a large number of presumptive hemangiosarcoma cases in companion dogs evaluated at primary care veterinary facilities in the United Kingdom. The authors have built a rigorous statistical model to evaluate the variables they have selected to determine contributing factors to survival of dogs with HSA in these primary practices. However, not quite 35% of the cases are confirmed by histopathology and an unknown proportion are confirmed by cytology. Nearly half of the dogs were euthanized at presentation on day 0. The overall median survival time was 9 days and the vast majority of dogs died from euthanasia rather than disease with only a small proportion receiving full care in the form of surgery and chemotherapy. In the end, this manuscript is a description of the euthanasia practices of veterinary general practitioners in the UK with a clear bias against treating hemangiosarcoma aggressively. The applicability in the US or other parts of the world is very unclear.

The manuscript is ponderously long, diluting the message of the manuscript. The greatest flaw of the paper is that the authors have evaluated objective patient factors to determine the contributors to survival from HSA, when the fact that half of the patients were euthanized immediately and half of the rest within nine days makes it unlikely that any of the patient characteristics were the primary driving factor in the euthanasia decision. The fact that histologic confirmation of the disease was not required for the study is a confounding factor, as other diseases that are indistinguishable from HSA using clinical tests could have had better to excellent prognosis and were clearly not considered. For example, how many cases of splenic hematoma were diagnosed in the same year? That would be an interesting evaluation to determine the strength of the bias towards HSA as the diagnosis in hemoabdomen or splenic mass in this group of practitioners. Similarly, adding other splenic tumor diagnoses to the analysis would help evaluate the extent to which the population of veterinarians is simply biased towards HSA as a diagnosis and against its treatment.

*Thank you to the reviewer for raising these interesting points. Due to the reviewer’s concern about clinically diagnosed versus laboratory-confirmed diagnoses of haemangiosarcoma having very different prognoses we have now examined survival time and created a separated survival model of those cases with a histologically confirmed diagnosis. Many of the findings in this model are similar as to those in the presumptive model (tumour size, surgery, location) and as such we believe the findings from both models together complement each other in this paper to explain haemangiosarcoma (clinically diagnosed and confirmed cases) survival in first opinion practice. This “clinically diagnosed” clarification has been amended throughout the manuscript. Due to the limited sensitivity of cytology in diagnosing sarcomas, particularly haemangiosarcoma, and the reliance on histopathological evaluation of surgically obtained biopsies for a definitive diagnosis, studies that include only histologically confirmed cases are inherently biased toward dogs with localised or loco-regional disease. This is because surgical excision is more likely to be pursued in cases where the tumour is at an early stage and the procedure is clinically justifiable. Consequently, such studies may underrepresent dogs with advanced or metastatic haemangiosarcoma, in whom invasive diagnostic procedures are either contraindicated (due to high risk of bleeding) or provide limited clinical benefit, thereby skewing the population toward earlier disease stages. Also in the oncology referral setting, dogs presenting with widespread metastatic disease will be presumptively diagnosed and treated for haemangiosarcoma in the absence of histopathology based on signalment, clinical presentation and th*ese *biases of cases to only those confirmed would therefore lead to falsely high survival rates and potentially different factors associated with disease than what is truly seen in first opinion practice in the UK.*

*Whilst the reviewer considers that patient characteristics are not the primary driving factor in the euthanasia decision, we would consider these demographic factors are relevant. For example, owners of older dogs or giant breed dogs could be much more likely to euthanise at presentation due to perceptions of a short life expectancy remaining.*

*The generalisability (or potential lack of) to other countries in the world has been expanded on in discussion. The following lines have been added or modified as a result of these comments:*

**Lines 1-3: Survival time and prognostic factors in dogs clinically diagnosed with haemangiosarcoma in UK first opinion practice**

**Line 32: Dogs clinically diagnosed with haemangiosarcoma in first-opinion practice in 2019 were**

**Lines 42-46: Of dogs with a histopathological diagnosis of haemangiosarcoma, overall MST was longer at 105 days (95% CI 84-133 days) and additionally, location-specific MST were longer. For both clinically diagnosed cases and histopathologically confirmed cases, increasing tumour size was associated with increased hazard of death while cutaneous location and surgery were associated with reduced hazard of death.**

**Line 49-52: However, a common prevailing view of extremely poor prognosis for haemangiosarcoma could be promoting frequent euthanasia at presentation and therefore leading to a self-fulfilling prophecy and low survival times. Further exploration of the potential effect of perceived prognosis is warranted.**

**Line 133-137: A haemangiosarcoma case was defined as a dog with evidence of a final clinical diagnosis (with or without histopathological confirmation) of haemangiosarcoma in the EHRs. As not all cases underwent a definitive histopathological diagnosis, the diagnosis of haemangiosarcoma was considered a clinical diagnosis in some dogs.**

**Line 145: confirmed diagnosis. Haemangiosarcoma cases had additional clinical information extracted**

**Line 197-203: Two separate survival analyses were undertaken using Cox proportional hazards regression modelling. In the first model, all dogs with ≥1 day survival after diagnosis (n=407) were considered to evaluate the hazard of death from both clinically diagnosed and histopathologically diagnosed haemangiosarcoma cases. In order to explore whether factors associated with hazard of death for dogs clinically diagnosed with haemangiosarcoma were consistent in lab confirmed haemangiosarcoma cases, a second model was built using only dogs with a histopathological diagnosis of haemangiosarcoma (n=272).**

**Line 273: Survival time across all clinically diagnosed haemangiosarcoma cases ranged from 0-1789 days**

**Line 287: Figure 2 – Kaplan-Meier survival curve estimating the survival time of clinically diagnosed**

**Line 292-299: Of those dogs with a confirmed histopathological diagnosis of haemangiosarcoma (n=272), MST was 105 days (84-133 days), with a 1-year survival rate of of 28% (22-34%). For hepatic, splenic and cutaneous haemangiosarcoma locations, the MST was longer for those with a clinical diagnosis (hepatic MST: 79 days (95% CI 35-112 days), splenic MST: 80 days (95% CI 64-106 days), cutaneous MST: 248 days (95% CI 119-478 days)). The 1-year survival rate for these locations was: 7.2% (95% CI1.9-26.7%), 16% (95%CI 10.1-24.2%) and 43.2% (95% CI 34.8-53.6). The MST and 1-year survival rate for histopathologically confirmed cardiac haemangiosarcoma could not be calculated as there was only one case.**

**Line 346:** **Hazard for death with clinically diagnosed haemangiosarcoma in dogs that survive for ≥1 day after diagnosis**

**Line 379-412:** **Hazard for death with histopathologically confirmed haemangiosarcoma in dogs**

**Of the 62 variables examined in univariable analyses, 38 were liberally significant (LRT p-value <0.2). Variables describing clinical signs at presentation were not taken forward due to their lack of independence as factors, leaving 29 factors to be assessed in multivariable modelling (Table A4 in Supplementary material). Of these, 15 variables were potentially correlated which were assessed in non-nested models for inclusion.**

**In the final Cox proportional hazards regression model, seven variables were retained: whether a patient had any form of surgery, location of haemangiosarcoma, maximum tumour size, whether a patient visited a referral centre, whether soft tissue metastases were identified, dog genotype grouping and the corporate group the clinic belonged to (Figure 5). Corporate group was not significant but was forced into the model to better account for the structure of the data. Additionally, a frailty term of clinic ID was incorporated to further account for data structure. The overall model and individual independent variables were assessed for violation of proportional hazards. All individual variables and the overall model satisfied this assumption. The final model performed well, with a pseudo-R2 of 0.39 and a Concordance Index of 70.**

**Surgery, haemangiosarcoma location and size were consistent between this model of histopathologically diagnosed haemangiosarcoma cases and all haemangiosarcoma cases. Dogs that had surgery performed had a reduced hazard of death (HR=0.39,95%CI 0.25-0.61), when compared to those that did not. Dogs with cutaneous haemangiosarcoma had decreased hazard of death compared to splenic haemangiosarcoma cases (HR=0.60,95%CI 0.43-0.83). Larger tumour size was associated with an increased hazard of death, this hazard was greatest for tumours between 8-23cm when compared to 0.2-4.3cm tumours (HR=2.59, 95%CI 1.42-4.71). Visiting a referral centre was associated with a two-fold reduction in hazard of death (HR =0.46, 95% CI 0.31-0.69). Dogs of the genotype group 2 (primarily consisting of spaniel, hound and poodle breeds) had decreased hazard of death (HR=0.52, 95% CI 0.31-0.89) when compared to crossbred dogs (Figure 5).**

**Figure 5- Forest plot of final mixed effects multivariable Cox proportional hazards regression results for factors associated with time to death with haemangiosarcoma in cases histopathologically diagnosed in first-opinion practices in VetCompass in 2019 with ≥1 day of survival post diagnosis (n=272). Cases (%) column indicates the number of cases for each category in the model. HR indicates the hazard ratio for each category and its 95% confidence interval (CI) and p-value. The final model included a frailty term of Clinic ID.**

**Line 417-424: with a first-opinion level of diagnosis. This study identified a short MST overall for haemangiosarcoma (nine days) and for each main tumour location. In hazard regression modelling of dogs with ≥1 day of survival after diagnosis and dogs that had a histopathological diagnosis, dogs with haemangiosarcoma of a cutaneous location and dogs that had any surgical management performed had a reduced hazard of death. Increasing tumour size was associated with increased hazard of death. Males were also found to have increased hazard of death when both clinically diagnosed and laboratory confirmed haemangiosarcoma cases were modelled.**

**Line 445-448:** **When MSTs were calculated for cases with ≥1 day of survival after diagnosis or cases with a histologically confirmed diagnosis in this Vet Compass population, MSTs were similar to those reported in previous studies (7,10,31–33).**

**Lines 521-523:** **Both location and tumour size associations were consistent for both the model built with all clinically diagnosed cases and the model of only histologically diagnosed cases.**

**Male dogs had an increased hazard of death with haemangiosarcoma in the clinically diagnosed cases model.**

**Line 547-553: When histologically confirmed cases were examined, rather than variation in hazard for single dog breeds, dogs of the genotype group that consisted of spaniel, poodle, dachshund and beagle breeds were found to have a reduced hazard of death (27). These were not the same breeds identified to have increased survival by Nóbrega and colleagues (2019) (14). No breed associations were identified in the model of all haemangiosarcoma cases, nor with the survival time overall, in agreement with other existing literature (15,20).**

**Line 576-587:** **Given the variation in proportions of biopsies between haemangiosarcoma locations in this study, three-quarters of cutaneous haemangiosarcoma cases were biopsied here versus just 2 % and 28% of cardiac and splenic cases, it seems plausible that the severity of clinical presentation and the ability of first-opinion vets to perform haemangiosarcoma surgeries would have affected the uptake of biopsies. (1) Additionally, cytological diagnosis has limitations (such as blood contamination of samples) and an invasive biopsy may not be considered justifiable in patients with advanced disease (1). Furthermore, histopathology alone can be insufficient to differentiate splenic haemangiosarcoma from other sarcomas, requiring immunohistochemistry confirmation which has additional time and cost implications (1) Thus, if the study only included histologically diagnosed dogs, a large proportion of dogs would be excluded and survival would be biased to dogs with early stage haemangiosarcoma and longer survival times.**

**Lines 588-590: Euthanasia will undoubtedly have impacted survival risk factors. However, this study was interested in reporting the survival pattern overall and therefore both dogs euthanised due to the disease and dogs that died from the disease need to be included.**

**Line 604-608: Whilst these findings are likely applicable across wider UK first-opinion veterinary practices, it is unclear how applicable these findings are to other countries in the world. Factors such as breed genetic differences in other countries, access to diagnostics, standards of care and owner attitudes to euthanasia and treatment might vary between countries and lead to different associations than those seen here.**

**Lines 619-638:** **Additionally, a small prospective study by Stewart and colleagues (2020) found that dogs presenting with splenic mass rupture (both benign and malignant) had more favourable outcomes than existing literature informing views on prognosis would suggest (44). This highlights the potential role of prospective studies in exploring outcomes and euthanasia rates and reasons in haemangiosarcoma cases. Whilst it was not within the scope of this project to explore factors motivating owners to pursue euthanasia, it is likely that it was not only related to the tumour aggressive behaviour, but also other factors such as the severity of the clinical signs and perceived acute deterioration in quality of life, together with the age at presentation (adult to elderly for large breeds), probable presence of age-related comorbidities and treatment costs.**

**Future work could also attempt to identify a control population of other splenic tumours to evaluate whether the presumed haemangiosarcoma group truly has distinct survival and could improve the external validity of the study. However doing this has some challenges that would need to be taken into consideration: other neoplasms within the spleen are rare (only account for 25% of malignant splenic tumours) and include (aside from lymphoma) mainly other sarcomas (histiocytic sarcoma, leiomyosarcoma, fibrosarcoma, undifferentiated sarcomas and liposarcoma) for which survival data in the literature is scarce (4). Additionally, histologically these sarcomas can often be challenging to distinguish from haemangiosarcoma and immunohistochemistry may be needed to achieve a definitive diagnosis, which may not be commonly pursued in the general practice setting.**

**Line 646-649: The potential effect of perceived prognosis on patient euthanasia should be assessed further as prevailing views of an extremely poor prognosis may result in more frequent euthanasia at presentation and create a self-fulfilling prophecy and short survival times.**

Minor comments:
In the dogs eliminated for the 5 year age restriction, did any have a histologic diagnosis of HSA?

*None of these dogs had a histologic diagnosis of HSA. This has now been added into the manuscript for clarification.*

**Lines 250-251: None of the age-excluded (dogs <5 years old) had histopathology performed.**

The benefit of having "no clinical signs at presentation" is highly questionable, given that this represents only 28 dogs out of the population and the group is not better described to understand the exact presentation. Were these mostly cutaneous HSA dogs?

*We have cross-tabulated to check the main tumour locations for this group. Of these dogs, the majority are classified as “non-cutaneous” (23/28, 82%) – with 22 of these being splenic and 1 being “non-splenic visceral”. Incidental splenic masses could still be found in a work-up for other conditions and therefore this is still interesting to include as a variable as this subgroup of dogs might benefit from an earlier diagnosis. Due to these analyses and the potential for incidental masses to be found in work-ups we think it is still helpful to retain this variable the model to indicate an admittedly more minor presentation but one that would still be relevant and of interest to readers.*

**Lines 484-488 Perhaps expectedly, an absence of clinical signs at diagnosis was associated with increased odds of surviving longer than one day. It seemed plausible that these would be more likely to be cutaneous lesions as these would be less likely to have clinical manifestations, however most of these cases were actually splenic (n=22/28). Although the case numbers for this variable are small, this might be of clinical relevance to practitioners finding an incidental splenic mass on a work-up.**

Hazard of death increased as tumor size increased, yet at least one paper has suggested that larger masses are less likely to be HSA. Given the lack of biopsy confirmation, were other differentials considered and are the veterinarians aware that larger tumors might carry a better prognosis with surgical removal? Again, a control group of dogs with other splenic tumors might identify overall population bias when HSA is the top considered diagnosis.

*Thank you for raising this point. We have incorporated into the paper a histological diagnosis only survival model. Many of the variables remained consistent between models with all (presumptive and confirmed) and only confirmed haemangiosarcoma cases. Across both models, increasing tumour size was associated with increased hazard of death. We comment in the discussion expand the discussion of this now and comment with a study showing size not to be associated with malignancy in splenic masses.*

*Whilst adding in a control population could create a comparative framework to evaluate the clinically diagnosed haemangiosarcoma group survival, there are substantial considerations for this approach that make it less feasible. Other neoplasms of the spleen are rare and mostly include other sarcomas (histiocytic sarcoma, leiomyosarcoma, fibrosarcoma, undifferentiated sarcomas and liposarcoma) with minimal survival data available. These other sarcomas are histologically challenging to diagnose, often requiring immunohistochemistry in addition to histopathology, which leads to a further biased population as these are tests less likely to be pursued in first opinion populations. We have now discussed the possibility of a control population and it’s challenges in the discussion/future work sections.*

*The following lines have been amended:*

**Line 511-514: Tumour size has not consistently been found to be a predictor of splenic malignancy as there is overlap between benign (specially hematomas) and malignant lesions, as well as between malignant lesions (27). Tumour size may only be associated with prognosis for certain haemangiosarcoma locations.**

**Line 629-638: Future work could also attempt to identify a control population of other splenic tumours to evaluate whether the presumed haemangiosarcoma group truly has distinct survival and could improve the external validity of the study. However doing this has some challenges that would need to be taken into consideration: other neoplasms within the spleen are rare (only account for 25% of malignant splenic tumours) and include (aside from lymphoma) mainly other sarcomas (histiocytic sarcoma, leiomyosarcoma, fibrosarcoma, undifferentiated sarcomas and liposarcoma) for which survival data in the literature is scarce (4). Additionally, histologically these sarcomas can often be challenging to distinguish from haemangiosarcoma and immunohistochemistry may be needed to achieve a definitive diagnosis, which may not be commonly pursued in the general practice setting.**

Harmonizing the language around the hazard ratios would be very helpful. "Increase in hazard" of death is very clear, but "half the reduction in hazard of death" to describe a HR of 0.48 is confusing as it is almost a double negative. Consider "decrease in hazard of death" and list the HR.
*Thank you reviewer, we have made corrections across the document for the reduced hazard associations and agree the language is clearer to understand this way.*

**Line 363-365: Male dogs had an increased hazard of death (HR=1.32, 95%CI 1.05-1.65) compared to female dogs. Dogs with any surgery performed had a reduction in hazard of death when compared to those that didn’t**

**Line 368-9: Dogs with a cutaneous haemangiosarcoma had a decrease in hazard of death versus dogs with cardiac haemangiosarcoma**

The discussion of male sex and hazard of death is perhaps unnecessary as the dogs in question did not die of actual disease complications, but of euthanasia and typically quite early in the course of the treatment. The factors that lead UK veterinarians to not treat HSA effectively is perhaps a better focus of the manuscript.
The likelihood of a "common prevailing view of extremely poor prognosis" is discussed only in the discussion. This is perhaps the headline of the study.

*Thank you for highlighting the importance of discussing the poor prognosis earlier in the text. We have now incorporated it into the abstract and made this clearer in the conclusion also. It was beyond the scope of this project to explore euthanasia motivation factors but we have discussed some potential reasons and future work in our discussion now around this aspect.*

**Line 49-52: However, a common prevailing view of extremely poor prognosis for haemangiosarcoma could be promoting frequent euthanasia at presentation and therefore leading to a self-fulfilling prophecy and low survival times. Further exploration of the potential effect of perceived prognosis is warranted.**

**Line 554-555: Due to the very short survival times and high uptake of euthanasia in this study, it must be considered that these factors are not the primary drivers of survival time.**

**Line 619-628: Additionally, a small prospective study by Stewart and colleagues (2020) found that dogs presenting with splenic mass rupture (both benign and malignant) had more favourable outcomes than existing literature informing views on prognosis would suggest (44). This highlights the potential role of prospective studies in exploring outcomes and euthanasia rates and reasons in haemangiosarcoma cases. Whilst it was not within the scope of this project to explore factors motivating owners to pursue euthanasia, it is likely that it was not only related to the tumour aggressive behaviour, but also other factors such as the severity of the clinical signs and perceived acute deterioration in quality of life, together with the age at presentation (adult to elderly for large breeds), probable presence of age-related comorbidities and treatment costs.**

**Line 646-649: The potential effect of perceived prognosis on patient euthanasia should be assessed further as prevailing views of an extremely poor prognosis may result in more frequent euthanasia at presentation and create a self-fulfilling prophecy and short survival times.**

Reviewer #2: The manuscript entitled “Survival time and prognostic factors in dogs diagnosed with haemangiosarcoma in UK first opinion practice” uses a large database of patient records to look at survival and potential prognostic factors.
Overall this is an interesting well described study that adds to the literature. I do have some comments and suggestions.
Title: I feel this title is a bit misleading as these cases do not necessarily have a true diagnosis but are presumed to have this diagnosis in many cases. I recognize that in the discussion this is addressed but I think would be useful to change the title to include presumed hemangiosarcoma or something similar.

*We have amended the title to now read as: “Survival time and prognostic factors in dogs clinically diagnosed with haemangiosarcoma in UK first opinion practice”. We have also made it clearer about what a clinical diagnosis is throughout the manuscript and modified accordingly (these line corrections are above in response to Reviewer 1’s concerns about the terminology)*

**Line 1-3: Survival time and prognostic factors in dogs clinically diagnosed with haemangiosarcoma in UK first opinion practice**

Lines 143-145 – I am not sure how this was applied or what is meant. If no histopathology was obtained then these cases have hemangiosarcoma as a differential diagnosis and not actually a diagnosis of hemangiosarcoma.

*We agree this could be made clearer. With many VetCompass studies we define ‘final clinical diagnosis’ as it relates to how a first opinion practitioner would diagnose a case. Due to the critical nature of many haemangiosarcoma cases, biopsies would often not be feasible or desirable to an owner. In dogs with widespread metastases it would be difficult to justify the pursuit of a histopathological diagnosis. Therefore, if in the clinical records the vet records their final diagnosis as haemangiosarcoma (without it being part of a list of differentials), we would consider it as diagnosed clinically as haemangiosarcoma. However, we have amended the language to refer to the whole study population as ‘clinically diagnosed’ cases and then report separately on just those with a histological diagnosis. We have created a separate model of only histologically diagnosed dogs and factors associated with their survival, finding largely consistent associations with survival in both models.*

**Lines 1-3: Survival time and prognostic factors in dogs clinically diagnosed with haemangiosarcoma in UK first opinion practice**

**Line 32: Dogs clinically diagnosed with haemangiosarcoma in first-opinion practice in 2019 were**

**Lines 42-46: Of dogs with a histopathological diagnosis of haemangiosarcoma, overall MST was longer at 105 days (95% CI 84-133 days) and additionally, location-specific MST were longer. For both clinically diagnosed cases and histopathologically confirmed cases, increasing tumour size was associated with increased hazard of death while cutaneous location and surgery were associated with reduced hazard of death.**

**Line 49-52: However, a common prevailing view of extremely poor prognosis for haemangiosarcoma could be promoting frequent euthanasia at presentation and therefore leading to a self-fulfilling prophecy and low survival times. Further exploration of the potential effect of perceived prognosis is warranted.**

**Line 133-137: A haemangiosarcoma case was defined as a dog with evidence of a final clinical diagnosis (with or without histopathological confirmation) of haemangiosarcoma in the EHRs. As not all cases underwent a definitive histopathological diagnosis, the diagnosis of haemangiosarcoma was considered a clinical diagnosis in some dogs.**

**Line 145: confirmed diagnosis. Haemangiosarcoma cases had additional clinical information extracted**

**Line 197-203: Two separate survival analyses were undertaken using Cox proportional hazards regression modelling. In the first model, all dogs with ≥1 day survival after diagnosis (n=407) were considered to evaluate the hazard of death from both clinically diagnosed and histopathologically diagnosed haemangiosarcoma cases. In order to explore whether factors associated with hazard of death for dogs clinically diagnosed with haemangiosarcoma were consistent in lab confirmed haemangiosarcoma cases, a second model was built using only dogs with a histopathological diagnosis of haemangiosarcoma (n=272).**

**Line 273: Survival time across all clinically diagnosed haemangiosarcoma cases ranged from 0-1789 days**

**Line 287: Figure 2 – Kaplan-Meier survival curve estimating the survival time of clinically diagnosed**

**Line 292-299: Of those dogs with a confirmed histopathological diagnosis of haemangiosarcoma (n=272), MST was 105 days (84-133 days), with a 1-year survival rate of of 28% (22-34%). For hepatic, splenic and cutaneous haemangiosarcoma locations, the MST was longer for those with a clinical diagnosis (hepatic MST: 79 days (95% CI 35-112 days), splenic MST: 80 days (95% CI 64-106 days), cutaneous MST: 248 days (95% CI 119-478 days)). The 1-year survival rate for these locations was: 7.2% (95% CI1.9-26.7%), 16% (95%CI 10.1-24.2%) and 43.2% (95% CI 34.8-53.6). The MST and 1-year survival rate for histopathologically confirmed cardiac haemangiosarcoma could not be calculated as there was only one case.**

**Line 346:** **Hazard for death with clinically diagnosed haemangiosarcoma in dogs that survive for ≥1 day after diagnosis**

**Line 379-412:** **Hazard for death with histopathologically confirmed haemangiosarcoma in dogs**

**Of the 62 variables examined in univariable analyses, 38 were liberally significant (LRT p-value <0.2). Variables describing clinical signs at presentation were not taken forward due to their lack of independence as factors, leaving 29 factors to be assessed in multivariable modelling (Table A4 in Supplementary material). Of these, 15 variables were potentially correlated which were assessed in non-nested models for inclusion.**

**In the final Cox proportional hazards regression model, seven variables were retained: whether a patient had any form of surgery, location of haemangiosarcoma, maximum tumour size, whether a patient visited a referral centre, whether soft tissue metastases were identified, dog genotype grouping and the corporate group the clinic belonged to (Figure 5). Corporate group was not significant but was forced into the model to better account for the structure of the data. Additionally, a frailty term of clinic ID was incorporated to further account for data structure. The overall model and individual independent variables were assessed for violation of proportional hazards. All individual variables and the overall model satisfied this assumption. The final model performed well, with a pseudo-R2 of 0.39 and a Concordance Index of 70.**

**Surgery, haemangiosarcoma location and size were consistent between this model of histopathologically diagnosed haemangiosarcoma cases and all haemangiosarcoma cases. Dogs that had surgery performed had a reduced hazard of death (HR=0.39,95%CI 0.25-0.61), when compared to those that did not. Dogs with cutaneous haemangiosarcoma had decreased hazard of death compared to splenic haemangiosarcoma cases (HR=0.60,95%CI 0.43-0.83). Larger tumour size was associated with an increased hazard of death, this hazard was greatest for tumours between 8-23cm when compared to 0.2-4.3cm tumours (HR=2.59, 95%CI 1.42-4.71). Visiting a referral centre was associated with a two-fold reduction in hazard of death (HR =0.46, 95% CI 0.31-0.69). Dogs of the genotype group 2 (primarily consisting of spaniel, hound and poodle breeds) had decreased hazard of death (HR=0.52, 95% CI 0.31-0.89) when compared to crossbred dogs (Figure 5).**

**Figure 5- Forest plot of final mixed effects multivariable Cox proportional hazards regression results for factors associated with time to death with haemangiosarcoma in cases histopathologically diagnosed in first-opinion practices in VetCompass in 2019 with ≥1 day of survival post diagnosis (n=272). Cases (%) column indicates the number of cases for each category in the model. HR indicates the hazard ratio for each category and its 95% confidence interval (CI) and p-value. The final model included a frailty term of Clinic ID.**

**Line 417-424: with a first-opinion level of diagnosis. This study identified a short MST overall for haemangiosarcoma (nine days) and for each main tumour location. In hazard regression modelling of dogs with ≥1 day of survival after diagnosis and dogs that had a histopathological diagnosis, dogs with haemangiosarcoma of a cutaneous location and dogs that had any surgical management performed had a reduced hazard of death. Increasing tumour size was associated with increased hazard of death. Males were also found to have increased hazard of death when both clinically diagnosed and laboratory confirmed haemangiosarcoma cases were modelled.**

**Line 445-448:** **When MSTs were calculated for cases with ≥1 day of survival after diagnosis or cases with a histologically confirmed diagnosis in this Vet Compass population, MSTs were similar to those reported in previous studies (7,10,31–33).**

**Lines 519-520:** **Both location and tumour size associations were consistent for both the model built with all clinically diagnosed cases and the model of only histologically diagnosed cases.**

**Male dogs had an increased hazard of death with haemangiosarcoma in the clinically diagnosed cases model.**

**Line 547-553: When histologically confirmed cases were examined, rather than variation in hazard for single dog breeds, dogs of the genotype group that consisted of spaniel, poodle, dachshund and beagle breeds were found to have a reduced hazard of death (27). These were not the same breeds identified to have increased survival by Nóbrega and colleagues (2019) (14). No breed associations were identified in the model of all haemangiosarcoma cases, nor with the survival time overall, in agreement with other existing literature (15,20).**

**Line 576-587:** **Given the variation in proportions of biopsies between haemangiosarcoma locations in this study, three-quarters of cutaneous haemangiosarcoma cases were biopsied here versus just 2 % and 28% of cardiac and splenic cases, it seems plausible that the severity of clinical presentation and the ability of first-opinion vets to perform haemangiosarcoma surgeries would have affected the uptake of biopsies. (1) Additionally, cytological diagnosis has limitations (such as blood contamination of samples) and an invasive biopsy may not be considered justifiable in patients with advanced disease (1). Furthermore, histopathology alone can be insufficient to differentiate splenic haemangiosarcoma from other sarcomas, requiring immunohistochemistry confirmation which has additional time and cost implications (1) Thus, if the study only included histologically diagnosed dogs, a large proportion of dogs would be excluded and survival would be biased to dogs with early stage haemangiosarcoma and longer survival times.**

Line 145 – Again using confirmed here could be misleading if they did not have an actual diagnosis – would suggest changing to suspected or presumed.

*As with above, have now changed to clinically diagnosed throughout the manuscript.*

Was whether or not someone had insurance looked as a factor? It would be interesting to see if this affected whether a dog survived one day or more – ie was the dog euthanized on the suspected diagnosis if the people did not have insurance for their dog?
*Ideally we would get to include insurance in this analyses as there is no doubt insurance would play a role in case outcomes. However, it is unclear if the presence of insurance in the data is current or just an historical status meaning this data is potentially unreliable for a large proportion of dogs and therefore we have not included. We have put a lack of insurance data as a study limitation.*

**Line 600-603: Insurance status likely has an effect on both diagnosis and survival time. Unfortunately, these data are often not up-to-date in EHRs. Due to this, the relationship between insurance status and haemangiosarcoma cases was not explored.**

Do you know if dogs were euthanized on the table for those dogs not living past the first day – ie the vet opened the dpg and euthanized because it looked bad?

*This was not an aspect of haemangiosarcoma cases we were able to examine fully in our analyses for several reasons. We have recorded the number of dogs that had an exploratory laparotomy (n=47) and the number of dogs who were euthanised due to an inoperable tumour(n=69). Additionally, of the 29 dogs that had surgery and died/were euthanised on day 0, almost all were recorded to have ‘inoperable tumour/s’ (pre or perioperative) recorded as their cause of death and almost all of these dogs were euthanised (n=27/29). However, to determine precisely when euthanasia happened is not possible.*

**Line 613-617: From the clinical notes in this study, it was not possible to determine whether dogs that died on the day of presentation and had surgery (n=29) were euthanised intra-operatively or after exploratory surgery, only that the majority were recorded to have ‘inoperable tumour/s’ (n=26) and the majority of these were euthanised (n=27) rather than died (n=2) .**
Were cases under 5 removed if they had a histopathological diagnosis – if so how many of these were there?

*None of these dogs had a histologic diagnosis of HSA. This has now been added into the manuscript for clarification.*

**Lines 250-251: None of the age-excluded (dogs <5 years old) had histopathology performed.**


Minor points:
Line 88 retrievers and shepherds should probably be lower case type.

*Amended now*

**Line 89: Whilst certain breed types (such as retrievers and shepherds) are at increased risk of diagnosis**

Line 182 has two periods at the end of the sentence.

*We could not find this error in the manuscript*

Supplemental Table A2 – “Urirary” – typographical error

*Amended now*
