# Supplementary material for: Survival time and prognostic factors in dogs clinically diagnosed with haemangiosarcoma in UK first opinion practice
Source: PLoS One. 2025 Jun 6;20(6):e0316066. doi: 10.1371/journal.pone.0316066 (PMC12143555; doi:10.1371/journal.pone.0316066)
Supplement: Peer review history

# Author comment: Author response to Decision Letter 0

**DOI:** 10.1371/journal.pone.0316066.r001

4 Dec 2024

# Peer review: Decision Letter 0

**DOI:** 10.1371/journal.pone.0316066.r002

**Authors:** Simon Clegg

11 Mar 2025

PONE-D-24-56041Survival time and prognostic factors in dogs diagnosed with haemangiosarcoma in UK first opinion practicePLOS ONE

Dear Dr. Taylor,

Thank you for submitting your manuscript to PLOS ONE. After careful consideration, we feel that it has merit but does not fully meet PLOS ONE’s publication criteria as it currently stands. Therefore, we invite you to submit a revised version of the manuscript that addresses the points raised during the review process.

==============================

Many thanks for submitting your manuscript to PLOS One

It was reviewed by two experts in the field, and they have recommended some modifications be made prior to acceptance

I therefore invite you to make these changes and to write a response to reviewers which will expedite revision upon resubmission

I wish you the best of luck with your modifications

==============================

Please submit your revised manuscript by Apr 25 2025 11:59PM. If you will need more time than this to complete your revisions, please reply to this message or contact the journal office at plosone@plos.org . When you're ready to submit your revision, log on to https://www.editorialmanager.com/pone/ and select the 'Submissions Needing Revision' folder to locate your manuscript file.

Please include the following items when submitting your revised manuscript:

- A rebuttal letter that responds to each point raised by the academic editor and reviewer(s). You should upload this letter as a separate file labeled 'Response to Reviewers'.
- A marked-up copy of your manuscript that highlights changes made to the original version. You should upload this as a separate file labeled 'Revised Manuscript with Track Changes'.
- An unmarked version of your revised paper without tracked changes. You should upload this as a separate file labeled 'Manuscript'.

If you would like to make changes to your financial disclosure, please include your updated statement in your cover letter. Guidelines for resubmitting your figure files are available below the reviewer comments at the end of this letter.

If applicable, we recommend that you deposit your laboratory protocols in protocols.io to enhance the reproducibility of your results. Protocols.io assigns your protocol its own identifier (DOI) so that it can be cited independently in the future. For instructions see: https://journals.plos.org/plosone/s/submission-guidelines#loc-laboratory-protocols . Additionally, PLOS ONE offers an option for publishing peer-reviewed Lab Protocol articles, which describe protocols hosted on protocols.io. Read more information on sharing protocols at https://plos.org/protocols?utm_medium=editorial-email&utm_source=authorletters&utm_campaign=protocols .

We look forward to receiving your revised manuscript.

Kind regards,

Simon Clegg, PhD

Academic Editor

PLOS ONE

Journal requirements: 

When submitting your revision, we need you to address these additional requirements.

1. Please ensure that your manuscript meets PLOS ONE's style requirements, including those for file naming. The PLOS ONE style templates can be found at

https://journals.plos.org/plosone/s/file?id=wjVg/PLOSOne_formatting_sample_main_body.pdf and

https://journals.plos.org/plosone/s/file?id=ba62/PLOSOne_formatting_sample_title_authors_affiliations.pdf .    

2.  Thank you for stating the following in the Competing Interests section:

[JL is an employee of the Morris Animal Foundation and authors CT and GB work is funded by a grant from the Morris Animal Foundation.].

Please confirm that this does not alter your adherence to all PLOS ONE policies on sharing data and materials, by including the following statement: ""This does not alter our adherence to  PLOS ONE policies on sharing data and materials.” (as detailed online in our guide for authors http://journals.plos.org/plosone/s/competing-interests).  If there are restrictions on sharing of data and/or materials, please state these. Please note that we cannot proceed with consideration of your article until this information has been declared.

Please include your updated Competing Interests statement in your cover letter; we will change the online submission form on your behalf.

3. When completing the data availability statement of the submission form, you indicated that you will make your data available on acceptance. We strongly recommend all authors decide on a data sharing plan before acceptance, as the process can be lengthy and hold up publication timelines. Please note that, though access restrictions are acceptable now, your entire data will need to be made freely accessible if your manuscript is accepted for publication. This policy applies to all data except where public deposition would breach compliance with the protocol approved by your research ethics board. If you are unable to adhere to our open data policy, please kindly revise your statement to explain your reasoning and we will seek the editor's input on an exemption. Please be assured that, once you have provided your new statement, the assessment of your exemption will not hold up the peer review process.

4. Please include captions for your Supporting Information files at the end of your manuscript, and update any in-text citations to match accordingly. Please see our Supporting Information guidelines for more information: http://journals.plos.org/plosone/s/supporting-information.

[Note: HTML markup is below. Please do not edit.]

Reviewers' comments:

Reviewer's Responses to Questions

**Comments to the Author**

1. Is the manuscript technically sound, and do the data support the conclusions?

The manuscript must describe a technically sound piece of scientific research with data that supports the conclusions. Experiments must have been conducted rigorously, with appropriate controls, replication, and sample sizes. The conclusions must be drawn appropriately based on the data presented. 

Reviewer #1: No

Reviewer #2: Yes

**********

2. Has the statistical analysis been performed appropriately and rigorously? 

Reviewer #1: Yes

Reviewer #2: Yes

**********

3. Have the authors made all data underlying the findings in their manuscript fully available?

The PLOS Data policy requires authors to make all data underlying the findings described in their manuscript fully available without restriction, with rare exception (please refer to the Data Availability Statement in the manuscript PDF file). The data should be provided as part of the manuscript or its supporting information, or deposited to a public repository. For example, in addition to summary statistics, the data points behind means, medians and variance measures should be available. If there are restrictions on publicly sharing data—e.g. participant privacy or use of data from a third party—those must be specified.

Reviewer #1: Yes

Reviewer #2: Yes

**********

4. Is the manuscript presented in an intelligible fashion and written in standard English?

PLOS ONE does not copyedit accepted manuscripts, so the language in submitted articles must be clear, correct, and unambiguous. Any typographical or grammatical errors should be corrected at revision, so please note any specific errors here.

Reviewer #1: Yes

Reviewer #2: Yes

**********

5. Review Comments to the Author

Please use the space provided to explain your answers to the questions above. You may also include additional comments for the author, including concerns about dual publication, research ethics, or publication ethics. (Please upload your review as an attachment if it exceeds 20,000 characters)

Reviewer #1: This manuscript is an interesting evaluation of a large number of presumptive hemangiosarcoma cases in companion dogs evaluated at primary care veterinary facilities in the United Kingdom. The authors have built a rigorous statistical model to evaluate the variables they have selected to determine contributing factors to survival of dogs with HSA in these primary practices. However, not quite 35% of the cases are confirmed by histopathology and an unknown proportion are confirmed by cytology. Nearly half of the dogs were euthanized at presentation on day 0. The overall median survival time was 9 days and the vast majority of dogs died from euthanasia rather than disease with only a small proportion receiving full care in the form of surgery and chemotherapy. In the end, this manuscript is a description of the euthanasia practices of veterinary general practitioners in the UK with a clear bias against treating hemangiosarcoma aggressively. The applicability in the US or other parts of the world is very unclear.

The manuscript is ponderously long, diluting the message of the manuscript. The greatest flaw of the paper is that the authors have evaluated objective patient factors to determine the contributors to survival from HSA, when the fact that half of the patients were euthanized immediately and half of the rest within nine days makes it unlikely that any of the patient characteristics were the primary driving factor in the euthanasia decision. The fact that histologic confirmation of the disease was not required for the study is a confounding factor, as other diseases that are indistinguishable from HSA using clinical tests could have had better to excellent prognosis and were clearly not considered. For example, how many cases of splenic hematoma were diagnosed in the same year? That would be an interesting evaluation to determine the strength of the bias towards HSA as the diagnosis in hemoabdomen or splenic mass in this group of practitioners. Similarly, adding other splenic tumor diagnoses to the analysis would help evaluate the extent to which the population of veterinarians is simply biased towards HSA as a diagnosis and against its treatment.

Minor comments:

In the dogs eliminated for the 5 year age restriction, did any have a histologic diagnosis of HSA?

The benefit of having "no clinical signs at presentation" is highly questionable, given that this represents only 28 dogs out of the population and the group is not better described to understand the exact presentation. Were these mostly cutaneous HSA dogs?

Hazard of death increased as tumor size increased, yet at least one paper has suggested that larger masses are less likely to be HSA. Given the lack of biopsy confirmation, were other differentials considered and are the veterinarians aware that larger tumors might carry a better prognosis with surgical removal? Again, a control group of dogs with other splenic tumors might identify overall population bias when HSA is the top considered diagnosis.

Harmonizing the language around the hazard ratios would be very helpful. "Increase in hazard" of death is very clear, but "half the reduction in hazard of death" to describe a HR of 0.48 is confusing as it is almost a double negative. Consider "decrease in hazard of death" and list the HR.

The discussion of male sex and hazard of death is perhaps unnecessary as the dogs in question did not die of actual disease complications, but of euthanasia and typically quite early in the course of the treatment. The factors that lead UK veterinarians to not treat HSA effectively is perhaps a better focus of the manuscript.

The likelihood of a "common prevailing view of extremely poor prognosis" is discussed only in the discussion. This is perhaps the headline of the study.

Reviewer #2: The manuscript entitled “Survival time and prognostic factors in dogs diagnosed with haemangiosarcoma in UK first opinion practice” uses a large database of patient records to look at survival and potential prognostic factors.

Overall this is an interesting well described study that adds to the literature. I do have some comments and suggestions.

Title: I feel this title is a bit misleading as these cases do not necessarily have a true diagnosis but are presumed to have this diagnosis in many cases. I recognize that in the discussion this is addressed but I think would be useful to change the title to include presumed hemangiosarcoma or something similar.

Lines 143-145 – I am not sure how this was applied or what is meant. If no histopathology was obtained then these cases have hemangiosarcoma as a differential diagnosis and not actually a diagnosis of hemangiosarcoma.

Line 145 – Again using confirmed here could be misleading if they did not have an actual diagnosis – would suggest changing to suspected or presumed.

Was whether or not someone had insurance looked as a factor? It would be interesting to see if this affected whether a dog survived one day or more – ie was the dog euthanized on the suspected diagnosis if the people did not have insurance for their dog?

Do you know if dogs were euthanized on the table for those dogs not living past the first day – ie the vet opened the dpg and euthanized because it looked bad?

Were cases under 5 removed if they had a histopathological diagnosis – if so how many of these were there?

Minor points:

Line 88 retrievers and shepherds should probably be lower case type.

Line 182 has two periods at the end of the sentence.

Supplemental Table A2 – “Urirary” – typographical error

**********

6. PLOS authors have the option to publish the peer review history of their article (what does this mean? ). If published, this will include your full peer review and any attached files.

If you choose “no”, your identity will remain anonymous but your review may still be made public.

**Do you want your identity to be public for this peer review?** For information about this choice, including consent withdrawal, please see our Privacy Policy .

Reviewer #1: No

Reviewer #2: No

**********

[NOTE: If reviewer comments were submitted as an attachment file, they will be attached to this email and accessible via the submission site. Please log into your account, locate the manuscript record, and check for the action link "View Attachments". If this link does not appear, there are no attachment files.]

While revising your submission, please upload your figure files to the Preflight Analysis and Conversion Engine (PACE) digital diagnostic tool, https://pacev2.apexcovantage.com/ . PACE helps ensure that figures meet PLOS requirements. To use PACE, you must first register as a user. Registration is free. Then, login and navigate to the UPLOAD tab, where you will find detailed instructions on how to use the tool. If you encounter any issues or have any questions when using PACE, please email PLOS at figures@plos.org . Please note that Supporting Information files do not need this step.

# Author comment: Author response to Decision Letter 0

**DOI:** 10.1371/journal.pone.0316066.r003

15 Apr 2025

14th April 2025

RE: Manuscript ID: PONE-D-24-56041

Manuscript title: Survival time and prognostic factors in dogs diagnosed with haemangiosarcoma in UK first opinion practice

Updated manuscript title: Survival time and prognostic factors in dogs clinically diagnosed with haemangiosarcoma in UK first opinion practice

Dear Dr Clegg and reviewers,

Thank you for your advice on how best to revise this manuscript and for the helpful and constructive further feedback from the reviewers. We have addressed all comments from both reviewers, as outlined below, with implementation or justification of all. We are grateful for these suggestions and feel that they have substantially enhanced this paper. We hope that you agree that these changes ensure that the overall paper now meets the standard required for publication in PLOS One.

Reviewer comments appear in normal text followed by any author responses in italics and then the revised manuscript lines and wording in bold. The line numbers quoted refer to the tracked changes version of the re-submission. In the resubmitted manuscript changes are highlighted in yellow.

Once again, thank you to PLOS One and to your reviewers for your excellent advice and support.

Yours sincerely,

Collette Taylor

Reviewer #1: This manuscript is an interesting evaluation of a large number of presumptive hemangiosarcoma cases in companion dogs evaluated at primary care veterinary facilities in the United Kingdom. The authors have built a rigorous statistical model to evaluate the variables they have selected to determine contributing factors to survival of dogs with HSA in these primary practices. However, not quite 35% of the cases are confirmed by histopathology and an unknown proportion are confirmed by cytology. Nearly half of the dogs were euthanized at presentation on day 0. The overall median survival time was 9 days and the vast majority of dogs died from euthanasia rather than disease with only a small proportion receiving full care in the form of surgery and chemotherapy. In the end, this manuscript is a description of the euthanasia practices of veterinary general practitioners in the UK with a clear bias against treating hemangiosarcoma aggressively. The applicability in the US or other parts of the world is very unclear.

The manuscript is ponderously long, diluting the message of the manuscript. The greatest flaw of the paper is that the authors have evaluated objective patient factors to determine the contributors to survival from HSA, when the fact that half of the patients were euthanized immediately and half of the rest within nine days makes it unlikely that any of the patient characteristics were the primary driving factor in the euthanasia decision. The fact that histologic confirmation of the disease was not required for the study is a confounding factor, as other diseases that are indistinguishable from HSA using clinical tests could have had better to excellent prognosis and were clearly not considered. For example, how many cases of splenic hematoma were diagnosed in the same year? That would be an interesting evaluation to determine the strength of the bias towards HSA as the diagnosis in hemoabdomen or splenic mass in this group of practitioners. Similarly, adding other splenic tumor diagnoses to the analysis would help evaluate the extent to which the population of veterinarians is simply biased towards HSA as a diagnosis and against its treatment.

Thank you to the reviewer for raising these interesting points. Due to the reviewer’s concern about clinically diagnosed versus laboratory-confirmed diagnoses of haemangiosarcoma having very different prognoses we have now examined survival time and created a separated survival model of those cases with a histologically confirmed diagnosis. Many of the findings in this model are similar as to those in the presumptive model (tumour size, surgery, location) and as such we believe the findings from both models together complement each other in this paper to explain haemangiosarcoma (clinically diagnosed and confirmed cases) survival in first opinion practice. This “clinically diagnosed” clarification has been amended throughout the manuscript. Due to the limited sensitivity of cytology in diagnosing sarcomas, particularly haemangiosarcoma, and the reliance on histopathological evaluation of surgically obtained biopsies for a definitive diagnosis, studies that include only histologically confirmed cases are inherently biased toward dogs with localised or loco-regional disease. This is because surgical excision is more likely to be pursued in cases where the tumour is at an early stage and the procedure is clinically justifiable. Consequently, such studies may underrepresent dogs with advanced or metastatic haemangiosarcoma, in whom invasive diagnostic procedures are either contraindicated (due to high risk of bleeding) or provide limited clinical benefit, thereby skewing the population toward earlier disease stages. Also in the oncology referral setting, dogs presenting with widespread metastatic disease will be pressumptively diagnosed and treated for haemangiosarcoma in the absence of histopathology based on signalment, clinical presentation and these biases of cases to only those confirmed would therefore lead to falsely high survival rates and potentially different factors associated with disease than what is truly seen in first opinion practice in the UK.

Whilst the reviewer considers that patient characteristics are not the primary driving factor in the euthanasia decision, we would consider these demographic factors are relevant. For example, owners of older dogs or giant breed dogs could be much more likely to euthanise at presentation due to perceptions of a short life expectancy remaining.

The generalisability (or potential lack of) to other countries in the world has been expanded on in discussion. The following lines have been added or modified as a result of these comments:

Lines 1-3: Survival time and prognostic factors in dogs clinically diagnosed with haemangiosarcoma in UK first opinion practice

Line 32: Dogs clinically diagnosed with haemangiosarcoma in first-opinion practice in 2019 were

Lines 42-46: Of dogs with a histopathological diagnosis of haemangiosarcoma, overall MST was longer at 105 days (95% CI 84-133 days) and additionally, location-specific MST were longer. For both clinically diagnosed cases and histopathologically confirmed cases, increasing tumour size was associated with increased hazard of death while cutaneous location and surgery were associated with reduced hazard of death.

Line 49-52: However, a common prevailing view of extremely poor prognosis for haemangiosarcoma could be promoting frequent euthanasia at presentation and therefore leading to a self-fulfilling prophecy and low survival times. Further exploration of the potential effect of perceived prognosis is warranted.

Line 133-137: A haemangiosarcoma case was defined as a dog with evidence of a final clinical diagnosis (with or without histopathological confirmation) of haemangiosarcoma in the EHRs. As not all cases underwent a definitive histopathological diagnosis, the diagnosis of haemangiosarcoma was considered a clinical diagnosis in some dogs.

Line 145: confirmed diagnosis. Haemangiosarcoma cases had additional clinical information extracted

Line 197-203: Two separate survival analyses were undertaken using Cox proportional hazards regression modelling. In the first model, all dogs with ≥1 day survival after diagnosis (n=407) were considered to evaluate the hazard of death from both clinically diagnosed and histopathologically diagnosed haemangiosarcoma cases. In order to explore whether factors associated with hazard of death for dogs clinically diagnosed with haemangiosarcoma were consistent in lab confirmed haemangiosarcoma cases, a second model was built using only dogs with a histopathological diagnosis of haemangiosarcoma (n=272).

Line 273: Survival time across all clinically diagnosed haemangiosarcoma cases ranged from 0-1789 days

Line 287: Figure 2 – Kaplan-Meier survival curve estimating the survival time of clinically diagnosed

Line 292-299: Of those dogs with a confirmed histopathological diagnosis of haemangiosarcoma (n=272), MST was 105 days (84-133 days), with a 1-year survival rate of of 28% (22-34%). For hepatic, splenic and cutaneous haemangiosarcoma locations, the MST was longer for those with a clinical diagnosis (hepatic MST: 79 days (95% CI 35-112 days), splenic MST: 80 days (95% CI 64-106 days), cutaneous MST: 248 days (95% CI 119-478 days)). The 1-year survival rate for these locations was: 7.2% (95% CI1.9-26.7%), 16% (95%CI 10.1-24.2%) and 43.2% (95% CI 34.8-53.6). The MST and 1-year survival rate for histopathologically confirmed cardiac haemangiosarcoma could not be calculated as there was only one case.

Line 346: Hazard for death with clinically diagnosed haemangiosarcoma in dogs that survive for ≥1 day after diagnosis

Line 379-412: Hazard for death with histopathologically confirmed haemangiosarcoma in dogs

Of the 62 variables examined in univariable analyses, 38 were liberally significant (LRT p-value <0.2). Variables describing clinical signs at presentation were not taken forward due to their lack of independence as factors, leaving 29 factors to be assessed in multivariable modelling (Table A4 in Supplementary material). Of these, 15 variables were potentially correlated which were assessed in non-nested models for inclusion.

In the final Cox proportional hazards regression model, seven variables were retained: whether a patient had any form of surgery, location of haemangiosarcoma, maximum tumour size, whether a patient visited a referral centre, whether soft tissue metastases were identified, dog genotype grouping and the corporate group the clinic belonged to (Figure 5). Corporate group was not significant but was forced into the model to better account for the structure of the data. Additionally, a frailty term of clinic ID was incorporated to further account for data structure. The overall model and individual independent variables were assessed for violation of proportional hazards. All individual variables and the overall model satisfied this assumption. The final model performed well, with a pseudo-R2 of 0.39 and a Concordance Index of 70.

Surgery, haemangiosarcoma location and size were consistent between this model of histopathologically diagnosed haemangiosarcoma cases and all haemangiosarcoma cases. Dogs that had surgery performed had a reduced hazard of death (HR=0.39,95%CI 0.25-0.61), when compared to those that did not. Dogs with cutaneous haemangiosarcoma had decreased hazard of death compared to splenic haemangiosarcoma cases (HR=0.60,95%CI 0.43-0.83). Larger tumour size was associated with an increased hazard of death, this hazard was greatest for tumours between 8-23cm when compared to 0.2-4.3cm tumours (HR=2.59, 95%CI 1.42-4.71). Visiting a referral centre was associated with a two-fold reduction in hazard of death (HR =0.46, 95% CI 0.31-0.69). Dogs of the genotype group 2 (primarily consisting of spaniel, hound and poodle breeds) had decreased hazard of death (HR=0.52, 95% CI 0.31-0.89) when compared to crossbred dogs (Figure 5).

Figure 5- Forest plot of final mixed effects multivariable Cox proportional hazards regression results for factors associated with time to death with haemangiosarcoma in cases histopathologically diagnosed in first-opinion practices in VetCompass in 2019 with ≥1 day of survival post diagnosis (n=272). Cases (%) column indicates the number of cases for each category in the model. HR indicates the hazard ratio for each category and its 95% confidence interval (CI) and p-value. The final model included a frailty term of Clinic ID.

Line 417-424: with a first-opinion level of diagnosis. This study identified a short MST overall for haemangiosarcoma (nine days) and for each main tumour location. In hazard regression modelling of dogs with ≥1 day of survival after diagnosis and dogs that had a histopathological diagnosis, dogs with haemangiosarcoma of a cutaneous location and dogs that had any surgical management performed had a reduced hazard of death. Increasing tumour size was associated with increased hazard of death. Males were also found to have increased hazard of death when both clinically diagnosed and laboratory confirmed haemangiosarcoma cases were modelled.

Line 445-448: When MSTs were calculated for cases with ≥1 day of survival after diagnosis or cases with a histologically confirmed diagnosis in this Vet Compass population, MSTs were similar to those reported in previous studies (7,10,31–33).

Lines 521-523: Both location and tumour size associations were consistent for both the model built with all clinically diagnosed cases and the model of only histologically diagnosed cases.

Male dogs had an increased hazard of death with haemangiosarcoma in the clinically diagnosed cases model.

Line 547-553: When histologically confirmed cases were examined, rather than variation in hazard for single dog breeds, dogs of the genotype group that consisted of spaniel, poodle, dachshund and beagle breeds were found to have a reduced hazard of death (27). These were not the same breeds identified to have increased survival by Nóbrega and colleagues (2019) (14). No breed associations were identified in the model of all haemangiosarcoma cases, nor with the survival time overall, in agreement with other existing literature (15,20).

Line 576-587: Given the variation in proportions of biopsies between haemangiosarcoma locations in this study, three-quarters of cutaneous haemangiosarcoma cases were biopsied here versus just 2 % and 28% of cardiac and splenic cases, it seems plausible that the severity of clinical presentation and the ability of first-opinion vets to perform haemangiosarcoma surgeries would have affected the uptake of biopsies. (1) Additionally, cytological diagnosis has limitations (such as blood contamination of samples) and an invasive biopsy may not be considered justifiable in patients with advanced disease (1). Furthermore, histopathology alone can be insufficient to differentiate splenic haemangiosarcoma from other sarcomas, requiring immunohistochemistry confirmation which has additional time and cost implications (1) Thus, if the study only included histologically diagnosed dogs, a large proportion of dogs would be excluded and survival would be biased to dogs with early stage haemangiosarcoma and longer survival times.

Lines 588-590: Euthanasia will undoubtedly have impacted survival risk factors. However, this study was interested in reporting the survival pattern overall and therefore both dogs euthanised due to the disease and dogs that died from the disease need to be included.

Line 604-608: Whilst these findings are likely applicable across wider UK first-opinion veterinary practices, it is unclear how applicable these findings are to other countries in the world. Factors such as breed genetic differences in other countries, access to diagnostics, standards of care and owner attitudes to euthanasia and treatment might vary between countries and lead to different associations than those seen here.

Lines 619-638: Additionally, a small prospective study by Stewart and colleagues (2020) found that dogs presenting with splenic mass rupture (both benign and malignant) had more favourable outcomes than existing literature informing views on prognosis would suggest (44). This highlights the potential role of prospective studies in exploring outcomes and euthanasia rates and reasons in haemangiosarcoma cases. Whilst it was not within the scope of this project to explore factors motivating owners to pursue euthanasia, it is likely that it was not only related to the tumour aggressive behaviour, but also other factors such as the severity of the clinical signs and perceived acute deterioration in quality of life, together with the age at presentation (adult to elderly for large breeds), probable presence of age-related comorbidities and treatment costs.

Future work could also attempt to identify a control population of other splenic tumours to evaluate whether the presumed haemangiosarcoma group truly has distinct survival and could impro

Attachment Submitted filename: 250414_letter to editor and reviewers PLOS ONE.docx

# Editor report: Decision Letter 1

**DOI:** 10.1371/journal.pone.0316066.r004

**Authors:** Simon Clegg

28 Apr 2025

Survival time and prognostic factors in dogs clinically diagnosed with haemangiosarcoma in UK first opinion practice

PONE-D-24-56041R1

Dear Dr. Taylor,

We’re pleased to inform you that your manuscript has been judged scientifically suitable for publication and will be formally accepted for publication once it meets all outstanding technical requirements.

Within one week, you’ll receive an e-mail detailing the required amendments. When these have been addressed, you’ll receive a formal acceptance letter and your manuscript will be scheduled for publication.

An invoice will be generated when your article is formally accepted. Please note, if your institution has a publishing partnership with PLOS and your article meets the relevant criteria, all or part of your publication costs will be covered. Please make sure your user information is up-to-date by logging into Editorial Manager at Editorial Manager®  and clicking the ‘Update My Information' link at the top of the page. If you have any questions relating to publication charges, please contact our Author Billing department directly at authorbilling@plos.org.

If your institution or institutions have a press office, please notify them about your upcoming paper to help maximize its impact. If they’ll be preparing press materials, please inform our press team as soon as possible -- no later than 48 hours after receiving the formal acceptance. Your manuscript will remain under strict press embargo until 2 pm Eastern Time on the date of publication. For more information, please contact onepress@plos.org.

Kind regards,

Simon Clegg, PhD

Academic Editor

PLOS ONE

Additional Editor Comments:

I wish to thank the authors for addressing the reviewer comments.

The manuscript reads well and I am pleased to recommend it for publication

I wish you the very best for your future research

# Editor report: Acceptance letter

**DOI:** 10.1371/journal.pone.0316066.r005

**Authors:** Simon Clegg

PONE-D-24-56041R1

PLOS ONE

Dear Dr. Taylor,

I'm pleased to inform you that your manuscript has been deemed suitable for publication in PLOS ONE. Congratulations! Your manuscript is now being handed over to our production team.

At this stage, our production department will prepare your paper for publication. This includes ensuring the following:

* All references, tables, and figures are properly cited

* All relevant supporting information is included in the manuscript submission,

* There are no issues that prevent the paper from being properly typeset

You will receive further instructions from the production team, including instructions on how to review your proof when it is ready. Please keep in mind that we are working through a large volume of accepted articles, so please give us a few days to review your paper and let you know the next and final steps.

Lastly, if your institution or institutions have a press office, please let them know about your upcoming paper now to help maximize its impact. If they'll be preparing press materials, please inform our press team within the next 48 hours. Your manuscript will remain under strict press embargo until 2 pm Eastern Time on the date of publication. For more information, please contact onepress@plos.org.

If we can help with anything else, please email us at customercare@plos.org.

Thank you for submitting your work to PLOS ONE and supporting open access.

Kind regards,

PLOS ONE Editorial Office Staff

on behalf of

Dr. Simon Clegg

Academic Editor

PLOS ONE
